# Repressive Cytosine Methylation is a Marker of Viral Gene Transfer Across Divergent Eukaryotes

**DOI:** 10.1093/molbev/msaf176

**Published:** 2025-07-25

**Authors:** Luke A Sarre, Giselle Azucena Gastellou Peralta, Pedro Romero Charria, Vladimir Ovchinnikov, Alex de Mendoza

**Affiliations:** School of Biological and Behavioural Sciences, Queen Mary University of London, London, UK; Centre for Epigenetics, Queen Mary University of London, London, UK; School of Biological and Behavioural Sciences, Queen Mary University of London, London, UK; Centre for Epigenetics, Queen Mary University of London, London, UK; School of Biological and Behavioural Sciences, Queen Mary University of London, London, UK; Centre for Epigenetics, Queen Mary University of London, London, UK; School of Biological and Behavioural Sciences, Queen Mary University of London, London, UK; Centre for Epigenetics, Queen Mary University of London, London, UK; School of Biological and Behavioural Sciences, Queen Mary University of London, London, UK; Centre for Epigenetics, Queen Mary University of London, London, UK

**Keywords:** epigenetics, DNA methylation, lateral gene transfer, viral endogenization, eukaryotes, heterochromatin

## Abstract

Cytosine DNA methylation patterns vary widely across eukaryotes, with its ancestral roles being understood to have included both transposable element (TE) silencing and host gene regulation. To further explore these claims, in this study, we reevaluate the evolutionary origins of DNA methyltransferases and characterize the roles of cytosine methylation on underexplored lineages, including the amoebozoan *Acanthamoeba castellanii*, the glaucophyte *Cyanophora paradoxa*, and the heterolobosean *Naegleria gruberi*. Our analysis of DNA methyltransferase evolution reveals a rich ancestral eukaryotic repertoire, with several eukaryotic lineages likely subsequently acquiring enzymes through lateral gene transfer (LGT). In the three species examined, DNA methylation is enriched on young TEs and silenced genes, suggesting an ancestral repressive function, without the transcription-linked gene body methylation of plants and animals. Consistent with this link with silencing, methylated genomic regions co-localize with heterochromatin marks, including H3K9me3 and H3K27me3. Notably, the closest homologs of many of the silenced, methylated genes in diverse eukaryotes belong to viruses, including giant viruses. Given the widespread occurrence of this pattern across diverse eukaryotic groups, we propose that cytosine methylation was a silencing mechanism originally acquired from bacterial donors, which was used to mitigate the expression of both transposable and viral elements, and that this function may persist in creating a permissive atmosphere for LGT in diverse eukaryotic lineages. These findings further highlight the importance of epigenetic information to annotate eukaryotic genomes, as it helps delimit potentially adaptive LGTs from silenced parasitic elements.

## Introduction

5-methylcytosine (5mC) is a widespread base modification in eukaryotes, which has evolved divergent patterns and functions across different lineages (Schmitz et al. 2019; de Mendoza et al. 2020; Sarkies 2022). In plants and animals, 5mC typically accumulates along gene bodies of constitutively expressed genes, with minimal methylation in promoter regions—a pattern referred to as Gene Body Methylation (GBM) (Feng et al. 2010; Zemach et al. 2010; Bewick and Schmitz 2017). In contrast, fungi and many algal groups predominantly accumulate 5mC in transposable elements (TEs) and transcriptionally silent DNA (Huff and Zilberman 2014; Bewick et al. 2019; Hoguin et al. 2023). Interestingly, plants and certain invertebrates possess both GBM and TE methylation (de Mendoza et al. 2019b; Lewis et al. 2020), leading to the hypothesis that both functions may have been present in the last common ancestor of eukaryotes (Zemach and Zilberman 2010; Yi 2024).

Supporting this hypothesis, DNA methyltransferases (DNMTs), which catalyze 5mC, are conserved across plants and animals (Law and Jacobsen 2010). DNMT1 orthologues, known as MET1 in plants, mediate maintenance methylation at CpG dinucleotides, while DNMT3 enzymes catalyze de novo methylation (Lyko 2018). Plants also encode additional DNMT1 paralogues in the Chromomethylases (CMT), which methylate CHG trinucleotides (H = A, T, C); and DNMT3 paralogues in the Domains Rearranged Methylases (DRM) that targets the CHH context, with both CHG and CHH methylation contexts predominantly associated to silencing (Bewick et al. 2017). In contrast, fungi and algal groups lacking GBM also typically lack DNMT3 (Huff and Zilberman 2014; Bewick et al. 2019), highlighting a potential connection between the presence of DNMT3 and GBM. It is however, noteworthy that GBM in plants does not directly depend on DNMT3 orthologues, as these are restricted to CHH methylation while GBM is limited to the CG dinucleotides, whereas animal DNMT3 enzymes are the main drivers of GBM through the targeting of the PWWP domain to H3K36me3 marked gene bodies.

Recent work identified that the unicellular ichthyosporean *Amoebidium* encodes both DNMT1 and DNMT3, and exhibits a GBM-like methylation pattern (Sarre et al. 2024). This raises the intriguing possibility that unicellular eukaryotes with both DNMT1 and DNMT3 could reflect the origins of GBM. However, this hypothesis remains largely unexplored. Complicating matters, the distribution of DNMTs across eukaryotes is highly dynamic, with multiple instances of DNMT loss and diversification (Ponger and Li 2005; Huff and Zilberman 2014; de Mendoza et al. 2018; Hoguin et al. 2023). Moreover, the evolutionary origins of eukaryotic DNMTs remain unclear. Unlike other core chromatin components, DNMTs are not monophyletic and lack archaeal ancestry. Instead, they appear to have arisen from ancient lateral gene transfer (LGT) events from bacteria to early eukaryotes (Jurkowski and Jeltsch 2011).

While the role of 5mC in silencing TEs is well-established, 5mC also plays a role in silencing viral endogenisation events (Stuhlmann et al. 1981; Sarre et al. 2024; Blais et al. 2025). This includes the endogenisation of giant viruses, whose insertions can be orders of magnitude larger than typical retrotransposons or endogenous retroviruses, encoding hundreds of genes and making them potentially harder to co-exist with native eukaryotic DNA (Moniruzzaman et al. 2020). So far, reports of giant virus endogenization marked by 5mC are limited to the moss *Physcomitrium patens*, the ichthyosporean *Amoebidium* and the amoebozoan *Acanthamoeba castellanii* (Maumus et al. 2014; Lang et al. 2018; Sarre et al. 2024; Blais et al. 2025). Given that virus-to-eukaryote LGT has occurred repeatedly throughout eukaryotic evolution (Irwin et al. 2022), a comprehensive evaluation of the relationship between 5mC and viral gene integration across diverse eukaryotic groups remains missing. Addressing this is critical, as 5mC may have facilitated the controlled integration and domestication of viral DNA into host genomes.

To address these knowledge gaps, we present the most complete phylogenetic analysis of DNMTs to date, reconstructing the ancestral DNMT repertoire of the last eukaryotic common ancestor (LECA). Our findings reveal recurrent cases of DNMT acquisition via LGT. Additionally, we characterize the 5mC methylomes of three understudied species, demonstrating that the presence of DNMT1 and DNMT3 does not correlate with GBM. Finally, we show that in species where 5mC plays a silencing role, viral LGT genes—often of giant virus origin—are frequently enriched among methylated genes. This suggests that 5mC-mediated silencing of viral DNA may have been an ancestral function in early eukaryotes, facilitating the stepwise integration of viral sequences into eukaryotic genomes.

## Results

### Eukaryotic DNMT Repertoires are Shaped by a Mix of Ancestral Genes and Lineage-specific Orphan DNMTs

To reconstruct the evolutionary history of cytosine DNA methyltransferases (DNMTs) in eukaryotes, we compiled a large dataset encompassing all eukaryotic supergroups, along with bacterial out-groups from previous studies (de Mendoza et al. 2018; Richter et al. 2022). It has been proposed that eukaryotic DNMTs originated from multiple bacterial donors (Jurkowski and Jeltsch 2011), with little support for a single ancestral event followed by eukaryote-specific duplications. To reassess this hypothesis, we included DNMTs from giant viruses, such as those from the *Nucleocytoviricota* and Mirusviruses, since some core eukaryotic genes have been suggested to originate from, or be co-opted by, these viral lineages (Bernabeu et al. 2024; Karki et al. 2025). We also examined Asgardarchaeota archaeal genomes, as several eukaryotic-specific proteins have been traced to this group (Eme et al. 2017).

**Fig. 1.**
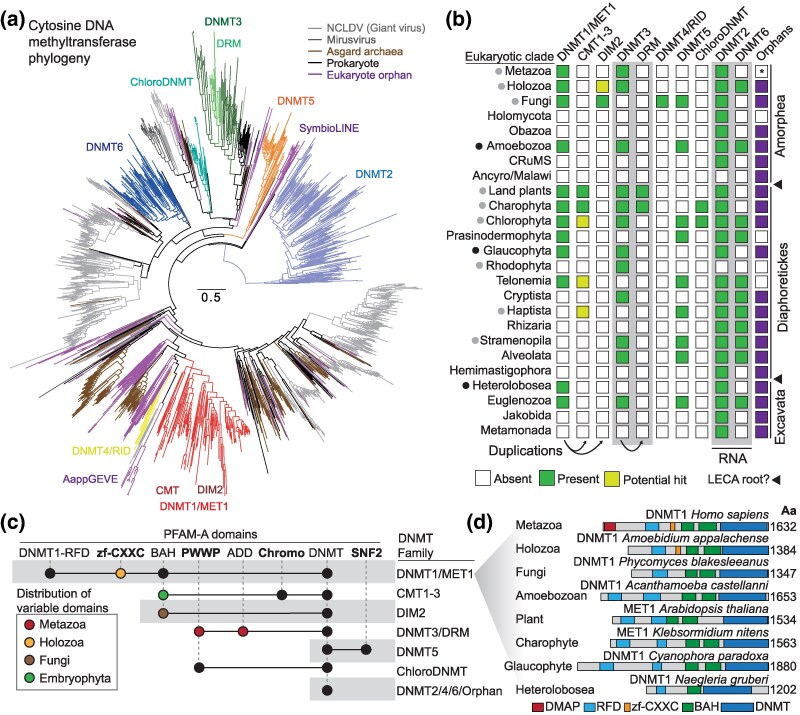
Evolutionary dynamics of DNMTs in the eukaryotes. a) Maximum likelihood phylogenetic tree of DNMTs, including eukaryotes, asgard archaea, viruses and bacteria. Each major eukaryotic DNMT family is highlighted with a color. Eukaryote orphan are eukaryotic sequences that do not cluster with any previously defined family. Gray branches depict viral sequences, brown branches depict Asgardarchaeota sequences, and black sequences depict bacteria. b) Distribution of eukaryotic DNMTs across the eukaryotes, based on phylogenetic profiling of panel a) and Dollo parsimony reconstruction of the ancestral state in each clade. Green cells depict presence, whereas yellow cells represent potential presence. Potential presence implies distinct domain architectures from “archetype” and low nodal branch supports. Black triangles highlight the two competing hypotheses regarding the root of extant eukaryotes. Raw data with per species DNMT repertoires can be found in supplementary fig. S1 and table S1, Supplementary Material online. An asterisk in animals indicates that few species are included in this analysis, therefore orphans are not ruled out. c) Protein domain architecture of the main eukaryotic DNMT families, as defined by PFAM domains. Each dot implies presence of the domain, but colored dots indicate that the domain is restricted to an eukaryotic lineage. d) Domain architectures of selected DNMT1 orthologues across major eukaryotic supergroups.

Our phylogenetic analysis revealed that eukaryotic DNMT groups are well-supported and comparable to previous classifications (Ponger and Li 2005; Huff and Zilberman 2014; Hoguin et al. 2023) (Fig. 1a). When charting the distribution of the DNMT1/2/3/5/6 clades across the eukaryotes, all were traceable to the LECA by applying Dollo parsimony ancestral state reconstruction (Fig. 1b, supplementary fig. S1a, Supplementary Material online). However, eukaryotic DNMT groups did not show close relationships with each other or with viral and archaeal DNMTs, suggesting the involvement of various bacterial donors or sequence change saturation limiting the resolution of deep branches in the DNMT tree (Fig. 1a). RNA-associated DNMT families DNMT2 and DNMT6 were not monophyletic, highlighting that substrate preference might have evolved independently. Importantly, we found no robust support that DNMTs from giant viruses or Asgardarchaeota form sister groups to eukaryotic DNMTs, implying that these groups might have contributed to the origins of eukaryotic DNMTs, but that this signal is not stronger than for other prokaryotic sources.

Among eukaryotic DNMTs, DNMT1 and DNMT5 displayed complex ancestral domain architectures (Fig. 1c), indicating early functional interactions with eukaryotic chromatin. For example, DNMT1 enzymes are characterized by the presence of Bromo Adjacent Homology (BAH) and Replication Foci Domains, both essential for maintaining 5mC during DNA replication (Lyko 2018). Even lineage-specific paralogues, such as plant CMT and fungal DIM2, retain the BAH domain (Fig. 1c). However, domain reconfiguration occurred throughout evolution, such as the addition of a zinc finger CXXC domain in holozoans or a Chromo domain in plant CMTs. Despite these changes, DNMT1 orthologues are remarkably conserved in both length and domain structure across all eukaryotic supergroups (Fig. 1d). Focused phylogeny of DNMT1 family revealed few potential cases of LGT, with sequences of *Telonema* and *Euglena* branching within a chlorophyte lineage (supplementary fig. S2, Supplementary Material online), which at least in the case of *Euglena* is consistent with the source of their secondary endosymbiosis (McFadden 2001).

DNMT5 orthologues in euglenozoans, algae, and fungi possess an additional C-terminal SNF2 ATPase domain, though this domain has been lost in some lineages, such as dinoflagellates and other algae. A focused phylogeny of DNMT5 orthologues did not provide strong evidence regarding eukaryote to eukaryote LGTs, despite its patchy distribution across the tree of life (supplementary fig. S3, Supplementary Material online), yet its complex branching pattern has been previously suggested to be the result of several lineage-specific duplications (Hoguin et al. 2023).

In contrast, DNMT3 orthologues show little domain conservation across eukaryotes. Animal DNMT3s contain PWWP and ADD domains, land plants possess a Domain of Unknown Function (Yaari et al. 2019), and protist and algal DNMT3s lack identifiable companion domains (Fig. 1c). The plant-specific paralogue of DNMT3, the DRM was found to be restricted to streptophytes, including many of the unicellular charophytes, suggesting that the RNA-mediated DNA methylation targeting evolved before the transition to land (Fig. 1b, supplementary fig. S3, Supplementary Material online). Then, some DNMT3 members are embedded within retrotransposon structures, including elements from both the LTR Copia and DIRS families, as previously reported in streptophytes and dinoflagellates (de Mendoza et al. 2018).

DNMT2 and DNMT6, which are associated with RNA modifications (Goll et al. 2006; Raddatz et al. 2013; Cuypers et al. 2020), consistently lack any companion domains, suggesting that DNMTs involved in DNA and chromatin regulation tend to acquire multifunctional domains, while those involved in RNA methylation do not.

Beyond the eukaryotic ancestral DNMT groups, we identified several lineages with restricted distributions (Fig. 1b). For example, DNMT4/RID appears to be confined to fungi, while a distinct lineage of DNMTs, which we term ChloroDNMTs, was found in chlorophytes and charophytes (Fig. 1b). This group often contains a PWWP domain, likely acquired independently of animal DNMT3s. Additionally, we observed several eukaryotic DNMTs that cluster into more lineage-specific clades which we name “orphans”, some of which had been previously identified as components of dinoflagellate LINE TEs (SymbioLINEs) (de Mendoza et al. 2018) or as part of giant virus endogenous elements in *Amoebidium* (Fig. 1a) (Sarre et al. 2024). While some of these orphan DNMTs may represent contaminants from nonaxenic cultures or poorly assembled genomes, we identified high-quality genomes that suggest these DNMTs were integrated through viral sources. For instance, the ameba *A. castellanii* (Neff strain) encodes a DNMT closely related to the DNMT of the giant virus *Pithovirus*. However, this viral DNMT is not expressed and is absent from the genome of *A. castellanii* C3 strain (Matthey-Doret et al. 2022), suggesting that it is unlikely to be functional. The widespread distribution of these orphan DNMTs across the eukaryotic dataset suggests that they are prone to being incorporated into eukaryotic genomes via LGT. The domain architectures of these DNMTs are generally sparse, lacking companion domains, and their dispersed positions in the phylogeny make it difficult to predict whether they function as DNA or RNA methyltransferases. Nevertheless, in our dataset, 12 species harbor only orphan DNMTs, having lost the ancestral DNMT1/3/5 enzymes (supplementary fig. S1, Supplementary Material online). This raises the possibility that, in some cases, 5mC-based methylation may be re-acquired through the incorporation of foreign DNMTs—a scenario reminiscent of recent findings in *Marchantia polymorpha* and *Adineta vaga*, where 4mC methyltransferases were independently acquired from bacteria (Rodriguez et al. 2022; Zhou et al. 2023; Walker et al. 2025).

Overall, our comprehensive dataset reveals that the LECA possessed a complex DNMT repertoire, including DNMT1, DNMT3, and DNMT5 as potential DNMT, and DNMT2 and DNMT6 as RNA methyltransferases (Fig. 1b). However, the distribution of these genes across modern eukaryotes is highly uneven, with DNMT2 being the least likely to undergo secondary loss, indicating its essential role in tRNA methylation. Importantly, 61% of species in our dataset (127) do not encode any DNMT with predicted DNA substrate, highlighting the pervasive role of secondary loss in 5mC evolution (supplementary fig. S1, Supplementary Material online). Ancestral and lineage-specific DNMTs are found in various combinations in extant species, underscoring the dynamic evolution of DNA methylation machinery across eukaryotic lineages.

### DNMT1/DNMT3-maintained Methylomes of *Acanthamoeba* and *Cyanophora* Lack GBM and are Enriched in Repressive 5mC

In plants and animals, DNMT1 and DNMT3 methyltransferases are responsible for both GBM and the silencing of TEs. These roles have been proposed as ancestral to eukaryotes. To test this further, we expanded DNA methylation data to key eukaryotic lineages that encode both DNMT1 and DNMT3, focusing on species positioned at critical points in the evolutionary tree. Specifically, we selected *A. castellanii* (Clarke et al. 2013), a free-living amoebozoan, which serves a sister group to the Opisthokonta (comprising animals and fungi), and the glaucophyte *Cyanophora paradoxa* (Price et al. 2012), the sister group to the Viridiplantae (comprising land plants and green algae) known for their chloroplasts with ancestral cyanobacteria features. The key amino acid residues required for 5mC activity were conserved in both DNMT1 and DNMT3 orthologues in these species (supplementary fig. S4, Supplementary Material online) (Gowher et al. 2006). In addition to their phylogenetic significance and conserved DNMT content, these species can be maintained in axenic culture, enabling the isolation of high-purity DNA and minimizing the risk of contamination.

**Fig. 2.**
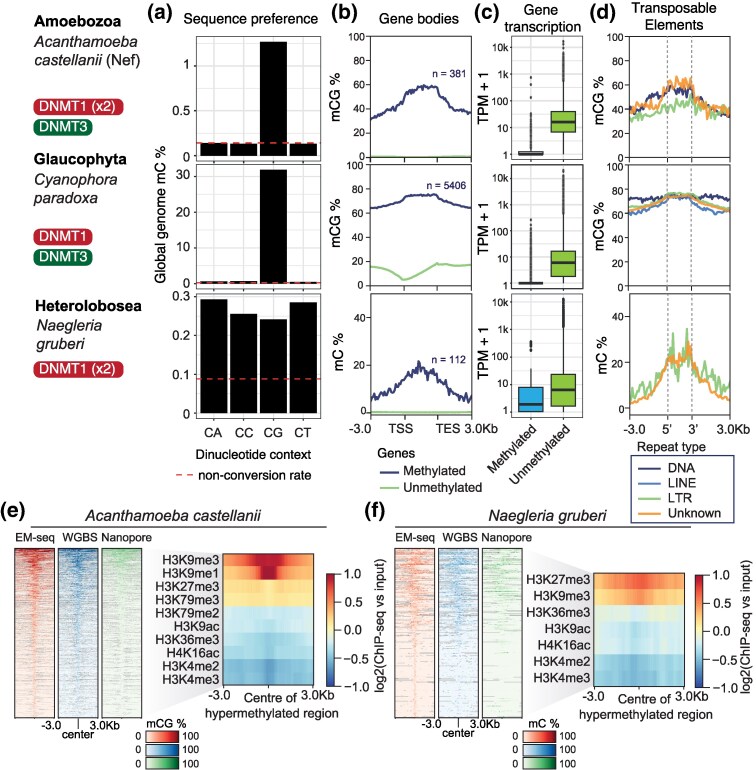
Cytosine methylome characteristics of three divergent eukaryotes. a) Global methylation levels at the four dinucleotide contexts as quantified with EM-seq in *A. castellanii*, *C. paradoxa* and *N. gruberi*. Dashed line indicates the cytosine methylation nonconversion rate (lambda genome control) in each EM-seq experiment. b) Average methylation levels on genes separated by their methylation status, with a threshold of ≥10% in *N. gruberi*, ≥ 20% in *A. castellanii*, and ≥50% in *C. paradoxa*. c) Distribution of transcriptional levels among methylated and unmethylated genes in each species, shown as the Transcript per Million+1, with a log10 transformed *y* axis. d) Average methylation levels on the three major classes of TEs (>500 bp) defined by RepeatModeler2, as well as unclassified repeats (Unknown), colored by legend below. Only methylated repeats (as per previous thresholds) with at least 20 methylated insertions are included. Heatmaps display methylation levels across hypermethylated regions of the *A. castellanii* e) and *N. gruberi* f) genomes, as measured by three independent technologies, alongside the average enrichment of histone posttranslational modifications, based on data from (Navarrete et al. 2025). 5mC levels in *A. castellanii* are for CG dinucleotides whereas in *N. gruberi* to all Cs.

Using Enzymatic Methyl-sequencing (EM-seq) (Vaisvila et al. 2021), we found that in *A. castellanii* (Neff strain) and *C. paradoxa*, methylation predominantly occurs at CpG dinucleotides (1.3% and 32%, respectively, Fig. 2a, supplementary fig. S5, Supplementary Material online), while non-CpG dinucleotides are below the nonconversion rate (0.2% to 0.3%), suggesting this signal is mostly false positives. In *C. paradoxa*, typical plant methylation sequence contexts (CHG and CHH) showed methylation levels near the nonconversion rate (CHG 0.73%, CHH 0.53%), suggesting that these contexts probably evolved later with the appearance of CMT and DRM methyltransferases in streptophytes.

In *A. castellanii*, few genes (381, 2.4%) were highly methylated (mCG ≥20%), and these genes were mostly transcriptionally silent (Fig. 2b and c), with methylation extending across the gene body and promoter—a pattern unlike plants and animals, where GBM correlates with transcriptional activity and promoters remain unmethylated. Methylated genes in A. castellanii were enriched in a whole range of functions, including kinase activity and DNA replication (supplementary fig. S6, Supplementary Material online), yet these are likely reflecting the composition of giant endogenous viruses (Blais et al. 2025). Methylation in *A. castellanii* was enriched in repetitive and intergenic regions, while introns and exons, which make up the majority of the genome (∼80%), exhibited low methylation (supplementary fig. S7a, Supplementary Material online). LINE and LTR retrotransposons were preferentially methylated, particularly younger copies (estimated using Kimura distances), except for a divergent group of LTRs (Fig. 2d, supplementary fig. S7b and c, Supplementary Material online). Overall, DNA transposons showed no similar enrichment, yet some inserts are highly methylated (Fig. 2d).

To investigate the relationship between 5mC and histone modifications in *A. castellanii*, we identified hypermethylated regions using a sliding window approach, which produced 966 windows spanning 3.4% of the genome. The methylation on these regions was highly consistent across different methylation detection methods, including EM-seq, WGBS, and Nanopore (Fig. 2e). We then mapped a recently generated ChIP-seq dataset for the Neff strain (Navarrete et al. 2025), which revealed that hypermethylated regions are enriched for repressive histone marks, most prominently H3K9me1/3, and to a lesser extent H3K27me3. In contrast, euchromatin histone marks associated with gene bodies (e.g. H3K36me3) or regulatory elements (e.g. H3K4me3) were depleted in these regions (Fig. 2e).

In *C. paradoxa*, we observed similar methylation patterns. Repetitive sequences were preferentially methylated, with both retrotransposons and DNA transposons showing high methylation levels (Fig. 2c), however repeat insertion age (estimated with Kimura distance) did not show a clear correlation with 5mC, which suggests that most repeats are effectively methylated in *C. paradoxa* (supplementary fig. S7d, Supplementary Material online). Moreover 5,406 genes (21%) showed elevated methylation (mCG >50%, Fig. 2b). Like *A. castellanii*, these methylated genes were primarily silent, indicating that methylation is associated with gene repression rather than activation (Fig. 2c). However, among the expressed genes in *C. paradoxa*, some degree of methylation accumulated towards the end of the gene body, a pattern so far unreported in other eukaryotes (Fig. 2b).

Overall, the data from *A. castellanii* and *C. paradoxa* resemble patterns in fungi and diatoms (Bewick et al. 2019; Hoguin et al. 2023; Ruggiero et al. 2023), showing that DNMT1 and DNMT3 are not predictive of GBM, and suggesting that these DNMTs were not linked to GBM in the eukaryotic ancestor. Instead, repressive 5mC, predominantly targeting repetitive and silent genes, likely represents the ancestral function of DNA methylation in eukaryotes.

### *Naegleria gruberi* Displays CX Methylation Maintained by DNMT1 Orthologues

The methylation data from amoebozoans and glaucophytes provides critical insights into the evolution of 5mC, but to fully understand early eukaryotic methylation patterns, it is essential to include discobans. Discobans occupy a key evolutionary position, either as a sister group to Diaphoretickes or to the rest of eukaryotes (Derelle et al. 2015; Al Jewari and Baldauf 2023; Williamson et al. 2025), making their methylation patterns valuable for identifying ancestral features. Among discobans and other members of the Excavata supergroup, only a few species have retained DNMTs, with euglenozoans and heteroloboseans being one of the few encoding DNMT1 or DNMT5 orthologues. We focused on the heterolobosean *N. gruberi*, a free-living ameboid, which contains a well-conserved DNMT1 enzyme (supplementary fig. S4, Supplementary Material online), has a high-quality genome assembly, and can be cultured axenically (Fritz-Laylin et al. 2010). Moreover, *N. gruberi* is well known for encoding a TET-like enzyme capable of oxidizing 5mC in vitro (Hashimoto et al. 2014; Pais et al. 2015), suggesting that this species may possess endogenous 5-hydroxymethylcytosine (5hmC) in its genome.

Using EM-seq on *N. gruberi*, we found low genome-wide methylation levels (0.28%), though these were still above the nonconversion rate (∼0.1%). Unlike *A. castellanii* and *C. paradoxa*, *N. gruberi* showed no preference for CpG dinucleotides—all four dinucleotide contexts were methylated at similar levels (Fig. 2a). The symmetry of CpG methylation was modest (*r* = 0.25), and few individual cytosine sites were fully methylated, suggesting variability in methylation from cell to cell (supplementary fig. S5, Supplementary Material online). To validate this pattern, we generated WGBS and called 5mC from our previously generated Nanopore datasets (Romero Charria et al. 2024). At base-pair resolution, 5mC calls between EM-seq and WGBS were highly congruent (correlation 0.838), whereas Nanopore showed a weaker correlation (0.387), although detection of non-CG methylation is known to be challenging for this technology (Kong et al. 2024) (supplementary fig. S8a, Supplementary Material online). Using a sliding window approach, we identified 447 hypermethylated regions in the *N. gruberi* genome, and all technologies consistently showed comparable methylation signal along that region (Fig. 2f, supplementary fig. S8b, Supplementary Material online), with both EM-seq and WGBS showing equivalent methylation levels in all four dinucleotides (Fig. 2f, supplementary fig. S8c and d, Supplementary Material online). This makes the *N. gruberi* methylome particularly interesting, as it indicates that despite possessing only two DNMT1 enzymes, the species can methylate non-CpG sites and likely exhibits de novo methylation activity, since the intermediate levels of cytosine methylation suggest a dynamic process of 5mC gain and loss, which could be linked to the presence of the TET-like enzymes. If this dual functionality and substrate plasticity of *N. gruberi* DNMT1 is ancestral or a lineage-specific variation is unclear.

To investigate whether 5hmC could explain the rapid turnover of 5mC levels in *N. gruberi*, we used Oxford Nanopore sequencing to directly detect this modification. While EM-seq and WGBS cannot distinguish between 5mC and 5hmC (Vaisvila et al. 2021), any potential 5hmC signal should be confined to regions where both methods detect cytosine methylation. Nanopore sequencing, in principle, allows for such discrimination. We analysed two Nanopore datasets generated with R9 and R10 pore technologies. R10 sequencing with Dorado enables the detection of 5hmC across all cytosine contexts, whereas R9 is limited to detecting 5hmC within CG dinucleotides. Upon examining Nanopore signals across the hypermethylated regions of the genome, we found no detectable 5hmC, and all modified cytosines were classified as 5mC (supplementary fig. S9, Supplementary Material online). It is important to note that 5hmC is typically found at very low levels (<1%), even in highly methylated vertebrate and sponge genomes (de Mendoza et al. 2019a), and that Nanopore sequencing has limited sensitivity for detecting low-frequency base modifications including 5hmC (Kong et al. 2024). Therefore, while we cannot rule out the presence of 5hmC in the *N. gruberi* genome, if present, it is likely a highly transient modification perhaps rapidly converted to further oxidized versions (5fC and 5caC) not recognized by Nanopore, or alternatively, the TET-like enzymes in this species may target thymine (Pais et al. 2015) or RNA as in *Drosophila* (Delatte et al. 2016).

Though 5mC was sparse in the *N. gruberi* genome, it accumulated disproportionately in repetitive regions (supplementary fig. S7a, Supplementary Material online). A small fraction of these repeats could be classified as known TEs, with both DNA transposons and retrotransposons showing higher methylation than the genomic background (Fig. 2d, supplementary fig. S7e, Supplementary Material online). Consistent with *A. castellanii*, younger TE insertions had higher methylation levels than older ones.

In terms of gene methylation, only 112 genes (0.6% of annotated genes) showed significant methylation (mC ≥10%), most of which were transcriptionally silent, yet with some exceptions (Fig. 2c, supplementary fig. S10, Supplementary Material online). Methylated genes were enriched in functions associated with cytoskeleton and translation blocking among others, yet a particular pathway was not evident (supplementary fig. S6, Supplementary Material online). This finding suggests that, similar to other eukaryotes, 5mC in *N. gruberi* primarily acts as a repressive mark, reinforcing the idea that ancestral eukaryotic methylation was largely restricted to repetitive elements and silenced genes.

Following the same approach used for *A. castellanii*, we mapped the previously published histone modification data to the hypermethylated regions of *N. gruberi* (Navarrete et al. 2025 ). This analysis revealed that 5mC-enriched regions coincide with H3K27me3 and H3K9me3, hallmarks of heterochromatin, while active chromatin marks were consistently depleted (Fig. 2f). These findings reinforce our conclusion that 5mC-marked regions are heterochromatic in *N. gruberi*, and support the hypothesis that the ancestral eukaryotic state involved the colocalization of DNA and histone-based silencing mechanisms.

### Cytidine Analogues Partially Deplete 5mC in *A. castellanii*

To directly assess the repressive function of 5mC in *A. castellanii* and *N. gruberi*, we treated cells with cytidine analogs known to incorporate into DNA and inhibit DNMTs, leading to passive 5mC dilution. We applied 5-Azacytidine, which is incorporated into both RNA and DNA in mammals, and its derivatives, Decitabine and Zebularine which are DNA-specific (Gowher and Jeltsch 2004). DMSO was used as a control, since it is required to solubilize the inhibitors. No growth defects were observed at any concentration. We selected concentrations that stayed below 1% DMSO, as higher levels affect *A. castellanii* growth (Siddiqui et al. 2016), yet were effective at demethylating based on concentrations previously used in the protist *Amoebidium* (Sarre et al. 2024).

After 3 d of treatment, we used EM-seq to profile methylation levels in both species. In *A. castellanii*, Decitabine and 5-Azacytidine markedly reduced global mCG levels by 29.5% and 23.4%, respectively, while Zebularine had no effect (supplementary fig. S11a and b, Supplementary Material online). In contrast, no inhibitors reduced methylation in *N. gruberi* (supplementary fig. S11e, Supplementary Material online), highlighting that cytidine analogs vary in efficacy across species, with some exhibiting the expected demethylation and others showing no response, which recapitulates previous observations in protists and animals (Guynes et al. 2024; Sarre et al. 2024). The exact reasons why different cytidine analogs may fail to induce demethylation across species remain unclear, highlighting the importance of validating their effects on DNA methylation before drawing conclusions from any observed phenotypes.

RNA-seq analysis revealed a broad transcriptional response in *N. gruberi* to cytidine analogs, with hundreds of genes differentially expressed across treatments, although only a fraction of methylated genes were affected, and these changed expression at lower fold changes than many unmethylated genes (supplementary fig. S11f and g, Supplementary Material online). Interestingly, both 5-Azacytidine and Decitabine led to comparable gene expression changes (supplementary fig. S11f, Supplementary Material online), predominantly affecting unmethylated genes associated with RNA export and protein polymerization (supplementary fig. S12, Supplementary Material online), suggesting that the cytidine analogs can exert broad transcriptional effects independent of DNA methylation. These cytotoxic effects are reminiscent of the transcriptional response to 5-Azacytidine in *Sphaeroforma arctica*, a species that lacks detectable 5mC (Sarre et al. 2024). This highlights the need to consider off-target or background effects when using cytidine analogs in nonmodel species, where their impact may not be limited to DNA demethylation.

In *A. castellanii*, three methylated genes were consistently upregulated upon demethylation treatment, ranking among the top differentially expressed genes sorted by transcriptional fold change (supplementary fig. S11c, Supplementary Material online). However, many unmethylated genes were differentially regulated with 5-Azacytidine or Decitabine, showing both up-regulation and downregulation upon treatment (supplementary fig. S11d, Supplementary Material online), suggesting widespread effects on the transcriptome beyond methylated genes, affecting various processes including cytoskeleton organization to metabolic functions (supplementary fig. S13, Supplementary Material online). In fact, the vast majority of the 381 methylated genes remained transcriptionally inactive. Among the repetitive elements that were upregulated, none were initially methylated. This indicates that cytidine analogs only partially inhibit methylation in *A. castellanii* and produce scarce transcriptional reactivation in methylated regions, while having substantial off-target effects. Since 5mC-methylated regions in *A. castellanii* are marked by both H3K9me3 and H3K27me3 (Fig. 2e), it is likely that loss of 5mC alone is insufficient for transcriptional reactivation. In both mammals and plants, loss of 5mC has been shown to lead to the recruitment of H3K27me3, suggesting a compensatory mechanism between these two repressive pathways (Walter et al. 2016 ; Déléris et al. 2021), which could also be the case in *A. castellanii*. In the case of viral genes and TEs, it is also possible that their promoter sequences have accumulated disabling mutations, or that essential trans-acting factors, such as specific transcription factors, are absent or not expressed under the conditions tested.

Among the methylated genes that were consistently upregulated in *A. castellanii*, all were part of giant virus endogenized regions (Blais et al. 2025). This suggests that these few genes from viral origin might be either especially sensitive to DNA methylation loss, or might be responsive to a nonspecific stress response caused by cytidine analogs.

### Genes From Viral Origins are Frequently Methylated in Eukaryotic Genomes

In most eukaryotic lineages with the notable exceptions of plants and animals, methylated genes are transcriptionally silent, belonging to TEs open reading frames, or part of hypermethylated giant virus endogenized elements (GEVEs). If 5mC is used as a mechanism to recognize and silence nonself DNA across eukaryotes, we wondered if the methylated fraction of genes would reveal the parts of the genome that have potential noneukaryotic origins across several lineages.

To test this, we used the data for *A. castellanii*, *C. paradoxa*, and *N. gruberi* and gathered whole genome methylation data from a subset of eukaryotes for which 5mC is associated with silencing, with some having reported GEVEs. These included streptophytes, diatoms and fungi (de Mendoza et al. 2018; Bewick et al. 2019; Griess et al. 2023; Hoguin et al. 2023; Sarre et al. 2024). Animals were not included in this analysis because most invertebrates accumulate 5mC predominantly in broadly transcribed housekeeping genes, which are highly conserved and unlikely to originate from LGT (de Mendoza et al. 2020). In vertebrates, DNA methylation is widespread, with the majority of genes methylated by default, making it difficult to detect targeted enrichment in specific gene categories. Similarly, in prasinophytes and haptophytes, 5mC is broadly distributed across gene bodies, where it is associated with nucleosome positioning rather than transcriptional silencing, further limiting its utility for assessing enrichment of specific functional classes (Huff and Zilberman 2014). For each of the selected species, we performed a DIAMOND search against NCBI nonredundant database, gathering the ten best hits for each gene and assigning potential taxonomic origin. In parallel, for each gene we classified them as methylated or unmethylated. The inclusion in the methylation category was tailored to each species particular methylome patterns, focusing on genes methylated in the sequence context that is associated with silencing in a given species (e.g. CHG methylation in streptophytes, CG in *A. castellanii*, or CX in *N. gruberi*).

**Fig. 3.**
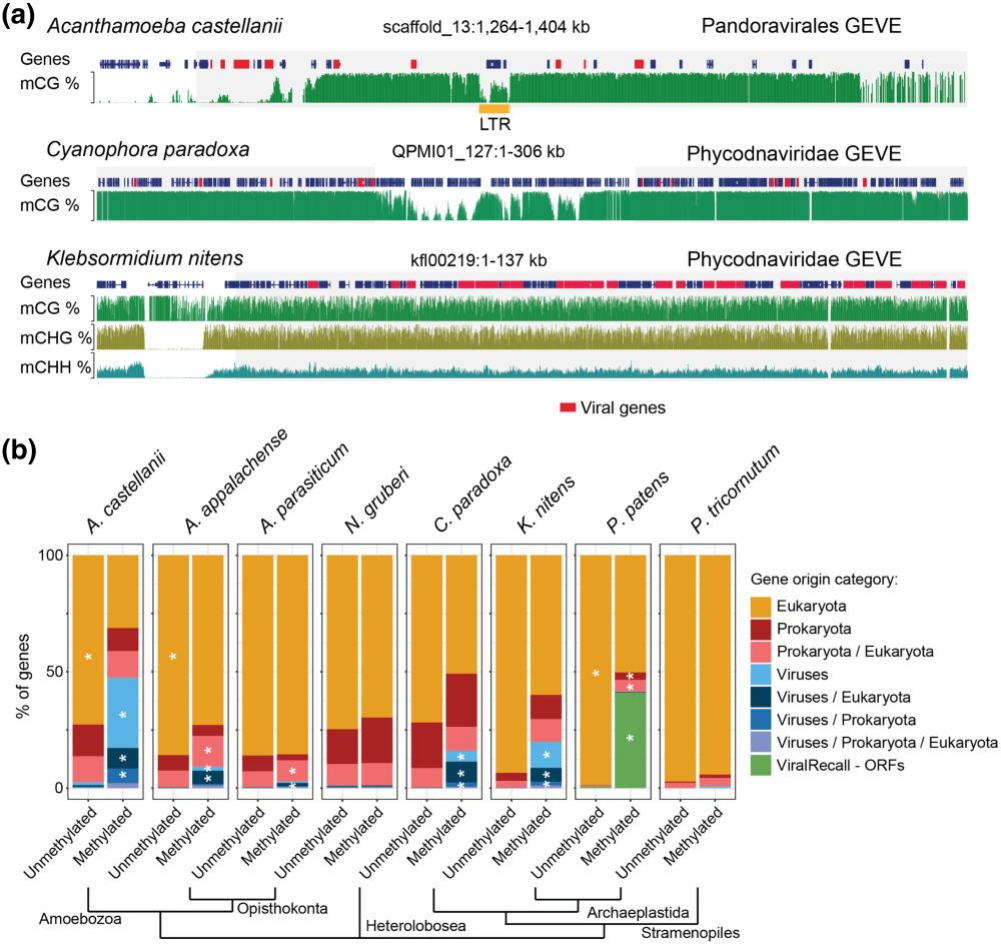
Viral endogenization is marked by cytosine methylation across distantly related eukaryotes. a) Genome browser examples of hypermethylated genomic regions belonging to Giant Viral Endogenous Elements in three distantly related eukaryotes. Methylation levels range from 0% to 100%, red genes have best NCBI nr hits against viral sequences, whereas blue genes have other ancestries. GEVE potential origin defined by gathering the taxonomy of all the viral hits in the region. b) Potential taxonomic origin of genes in eight distantly related eukaryotes, separated by their methylation status. Asterisks indicate significant enrichment of a given taxonomic category in the methylated or unmethylated state (fisher exact's test *P* < 0.001 two sided).

Crossing the information from potential taxonomic origin and methylation status, we observed that the methylated fraction tends to be enriched in genes with potential recent viral ancestry (Fig. 3a, supplementary fig. S14, Supplementary Material online). While *A. castellanii*, two *Amoebidium* species (*appalachense* and *parasiticum*), *C. paradoxa*, *Klebsormidium nitens*, and *Physcomitrium patens* showed strong methylation enrichments for the viral fraction of the genome, other species were less evident, for instance, we did not see an enrichment for viral genes in *N. gruberi* or its sister species *N. fowleri* (Liechti et al. 2019), and for the diatom *Phaeodactylum tricornutum*, where the only two genes in the genome with potential viral ancestry were methylated, but were insufficient to render a significant enrichment (Fig. 3a, supplementary fig. S14, Supplementary Material online). We further searched the taxonomic origin of methylated genes in several fungal species described to have islands of methylated genes (Bewick et al. 2019), and we did not find any support for those clusters of methylated genes having viral origins. In contrast, in most species in our dataset, methylated genes with eukaryotic origins were overwhelmingly genes with TE domains. Notably, the fraction of genes from potential prokaryotic LGTs were less likely to be enriched in the methylated fraction (Fig. 3a). This implies that these genes might be less prone to silencing by 5mC than genes from viral origin.

While the genes of recent viral ancestry mapped to a wide variety of viruses across the different species, the overwhelming majority were assigned to giant virus (*Nucleocytoviricota*) origin. When inspecting the genomic location of these genes, they tend to cluster in genomic neighborhoods. This approach revealed GEVEs in species for which these were not yet well described, including *C. paradoxa* or *K. nitens* (Fig. 3b). This expands the presence of GEVEs to branches of the tree of life for which these were not yet reported, and highlights how DNA methylation information can be very useful to determine the origin of important parts of the coding potential of a given genome.

## Discussion

**Fig. 4.**
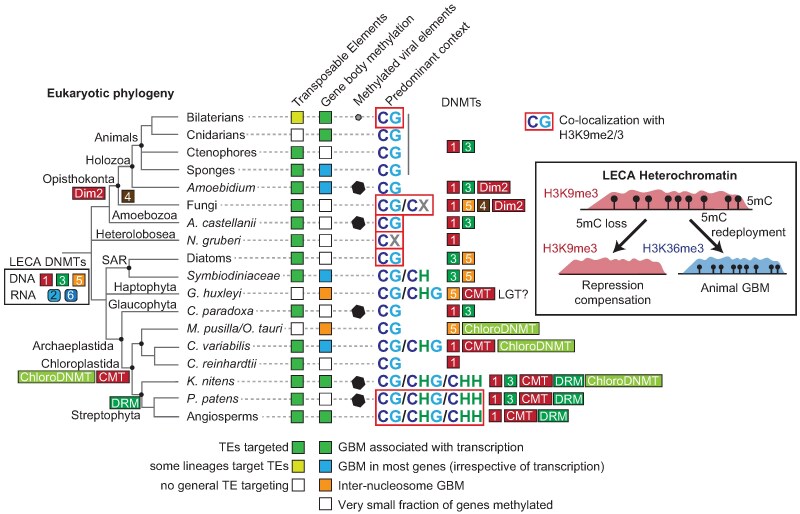
Diagram of 5mC pattern evolution across the eukaryotes. Phylogenetic tree of eukaryotes is based on current consensus branching patterns, and main DNMT families are shown as numbered colored squares, showing gains in the branches where they appear. Only lineages for which direct whole genome 5mC data is available are shown. TE and GBM are subdivided as different cases, specified in the legend below. Methylated viral elements are shown for confirmed cases, in the case of bilaterians, it is restricted (so far) to endogenous retrovirus in vertebrates. Predominant 5mC sequence context in the lineage, while some exceptions exist (e.g. CH methylation in the vertebrate neural system [de Mendoza et al. 2021]). Main DNMTs found in each lineage, although secondary loss occurs in many instances (e.g. many animals have lost DNMT1/3). Co-localization with H3K9me2/3 is highlighted for lineages in which both 5mC and H3K9me2/3 have been empirically measured. Among bilaterians, only vertebrates have been shown to exhibit co-localization of 5mC with H3K9me3.

Through profiling the 5mC methylomes of three eukaryotes at key phylogenetic positions of the eukaryotic tree of life, we demonstrate that 5mC silencing of TEs and viral-derived genes is widespread across eukaryotes. Yet, despite its potentially ancestral repressive function, 5mC patterns are highly dynamic and have diversified significantly over evolutionary time. In most eukaryotes, including the three species studied here, 5mC is predominantly associated with silenced genomic regions (de Mendoza et al. 2020). However, the GBM typical of plants and animals—where gene bodies are methylated but promoters remain unmethylated, often correlating with active transcription—is relatively rare and likely evolved convergently in these multicellular lineages (Fig. 4). An important difference between these two patterns is that in plants and animals, genes with GBM tend to be highly conserved (Sarda et al. 2012; Takuno and Gaut 2013; Guynes et al. 2024), whereas in species with 5mC playing exclusive repressive roles, methylated genes tend to be poorly conserved, even at the species level as seen across *Acanthamoeba* strains (Blais et al. 2025). Then, some unicellular lineages have large fractions of genes with methylation along the whole gene body, but this is unrelated to transcription, and presents many lineage-specific variations, such as default hypermethylation of promoters and gene bodies in dinoflagellates (de Mendoza et al. 2018; Li et al. 2024), or internucleosomal methylation in haptophytes and prasinophytes (Huff and Zilberman 2014). Given the wide range of genome sizes observed across species where 5mC functions as a repressive mark, the notion that epigenetic silencing inevitably leads to genome expansion is questionable (Zhou et al. 2020), implying that TE accumulation reflects a multifactorial process that cannot be solely attributed to one factor. The three species analysed in this study—*A. castellanii*, *N. gruberi*, and *C. paradoxa*—all possess genome sizes within the typical range for unicellular eukaryotes (40 to 100 Mb).

Despite the ancient conservation of DNMT families, predicting 5mC patterns just from DNMT repertoire remains elusive. Here we find that some DNMT3 encoding eukaryotes do not show GBM. Similarly, not all species whose methylomes are regulated by DNMT5, such as prasinophytes and haptophytes, present a distinctive internucleosomal 5mC pattern in gene bodies (Huff and Zilberman 2014). Species like the diatom *Phaeodactylum tricornutum* and the fungus *Cryptococcus neoformans* encode DNMT5 but exhibit a more restrictive methylation pattern limited to TEs (Huff and Zilberman 2014; Catania et al. 2020; Hoguin et al. 2023). This variation underscores the complexity and diversity of 5mC function across eukaryotes. Despite expanding our understanding, a vast portion of eukaryotic methylation remains uncharacterized, particularly among unicellular eukaryotic lineages. Advances in sequencing technology, particularly long-read methods like Oxford Nanopore and PacBio, which can natively detect 5mC, promise to rapidly expand our knowledge of methylation across new eukaryotic genomes. Here we show that Nanopore can be used to detect even noncanonical patterns as those of *N. gruberi*. From our survey of DNMTs, some particularly promising lineages that deserve attention are the Euglenids, *Prasinoderma*, charophytes or early branching brown algae encoding DNMT1 orthologues (Denoeud et al. 2024 ). However, a major challenge remains: the frequent loss of DNMTs in various eukaryotic groups. There is ample evidence that species only encoding RNA methyltransferases (DNMT2 or DNMT6) or lacking DNMTs, have undetectable 5mC levels (Raddatz et al. 2013; Cuypers et al. 2020; Kyger et al. 2021; Drewell et al. 2023; Sarre et al. 2024), which is important when considering 5mC profiling in new lineages, especially since most 5mC techniques have some level of false positives. This evolutionary trend toward a reduced DNMT repertoire suggests redundancy among DNMTs, as no extant species retains the full complement of DNMTs we hypothesize were present in the LECA (Fig. 4). For instance, the presence of the maintenance enzyme DNMT1 might render DNMT5 redundant, and vice versa. In stark contrast, the green lineage, particularly the streptophytes, exhibits a complex expansion of its DNMT repertoire, incorporating DRM and CMT methyltransferases, allowing for methylation in broader sequence contexts (CG, CHG, and CHH), often tied to small RNA-mediated defence mechanisms (Yaari et al. 2019). This complex DNMT repertoire is rarely lost in plants, hinting at a conserved evolutionary pressure for maintaining these mechanisms, which might have evolved from simpler patterns like those we observed in *C. paradoxa*. However, even in plants some exceptions exist, as loss of GBM has been reported in at least two angiosperms (Bewick et al. 2016; Muyle and Gaut 2019). However, total loss of 5mC is rampant across the eukaryotes, which is likely due to its potential cytotoxic effects and mutagenic potential (Pfeifer 2006; Rošić et al. 2018).

We also find that 5mC is a component of heterochromatin in both *N. gruberi* and *A. castellanii*, co-localizing with H3K9me3 and H3K27me3. A similar pattern has been reported in fungi, diatoms, vertebrates, and land plants (Déléris et al. 2021), suggesting that 5mC may have been associated with heterochromatin formation in early eukaryotes, functioning as part of a redundant silencing system alongside histone methylation (Fig. 4). This redundancy could help explain the recurrent loss of 5mC across eukaryotic lineages—its silencing role may be compensated for by histone-based repression. Given that 5mC is mutagenic and potentially cytotoxic (Duncan and Miller 1980; Rošić et al. 2018), it may have been more readily dispensable. This ancestral state has been modified in several lineages; for example, in invertebrates, 5mC predominantly co-localizes with H3K36me3 in active gene bodies (Schwaiger et al. 2014; Li et al. 2018), indicating a shift from heterochromatin to euchromatin. Interestingly, histone-mediated silencing systems, such as Polycomb repression and H3K9me3, have also been lost repeatedly across eukaryotes (Grau-Bové et al. 2022; de Potter et al. 2023), while activation-associated marks like H3K4me3 appear to be more consistently retained (Navarrete et al. 2025). Similarly, 6-methyladenine (6 mA), another ancestral base modification in eukaryotes, has also undergone multiple independent losses, further supporting functional redundancy as a recurring theme in chromatin evolution (Romero Charria et al. 2024). Importantly, 5mC loss may not be irreversible. We observe several species with orphan DNMTs in the absence of ancestral DNMT1/3/5, suggesting possible reacquisition. Recent studies have shown that foreign methyltransferases can be integrated into eukaryotic chromatin—for example, the independent acquisitions of a bacterial 4mC methyltransferases in *M. polymorpha* and *A. vaga* (Rodriguez et al. 2022; Zhou et al. 2023; Walker et al. 2025), or a prokaryotic 6 mA methyltransferase in early branching fungi (Lax et al. 2024). This indicates that base modifications can traverse domains of life through horizontal gene transfer. It is tempting to speculate that CMT-like DNMTs in haptophytes or the DNMT1 in *Euglena* may be remnants of eukaryote-to-eukaryote gene transfers during plastid acquisition via secondary endosymbiosis, further illustrating the dynamic evolution of DNA methylation systems across eukaryotes.

Importantly, we do not detect 5hmC signal in the genome of *N. gruberi*, which contrasts with the reports of a TET-like enzyme in this species (Hashimoto et al. 2014; Pais et al. 2015). This suggests that either the enzyme is not playing an active 5mC oxidation in vivo, or that this is very scarce and transient, and can be hardly detected. The *N. gruberi* TET-like enzyme was also shown to act on thymines (Pais et al. 2015), which might be the preferred substrate *in vivo*.

Beyond the role of 5mC in TE silencing, our findings also highlight its crucial role in the epigenetic repression of viral endogenizations. Viral sequences can represent a significant proportion of eukaryotic genomes, yet are rarely expressed. Our results suggest that, at least in species where 5mC acts as a repressive mark, viral-derived genes are frequently hypermethylated, likely as a mechanism to silence these potentially detrimental sequences. This raises intriguing evolutionary questions. While LGT is predicted to be rare in eukaryotes outside of major endosymbiotic events (Ku et al. 2015), frequent reports of viral-derived sequences in eukaryotic genomes nonetheless shows its importance (Irwin et al. 2022). Epigenetic silencing through 5mC provides a mechanism by which foreign DNA, especially viral sequences, may be integrated into host genomes without imposing immediate fitness costs (Sarre et al. 2024; Blais et al. 2025). This reconciles the apparent contradiction between the scarcity of conserved LGT in eukaryotes and the frequent detection of viral sequences in their genomes. However, viral endogenizations are not necessarily evolutionarily neutral. While most viral-derived genes are transcriptionally silent and under epigenetic control, some may occasionally gain a functional role, much like endogenous retroviruses in mammals, where viral-derived genes (e.g. syncytin) or regulatory elements have been co-opted for essential biological functions (Feschotte and Gilbert 2012). These viral domestication events, though relatively rare, highlight the potential for viral endogenizations to contribute to host adaptation (Johnson 2019). However, caution must be taken in ascribing function to endogenous viral DNA, as genome-wide LGT detection methods only relying on sequence comparisons and phylogenetic profiling can often overestimate functional significance of LGT by failing to account for the silenced state of many viral-derived genes. The methylated fraction of the genome is less likely to be conserved across species or isolates (Blais et al. 2025), contributing to the nonessential pangenome.

It is important to note that not all species retaining 5mC function exhibit giant endogenous viral elements. For example, *N. gruberi* has recently been shown to be infected by giant viruses (Arthofer et al. 2024), yet we find no clear evidence of large-scale endogenization in its genome. Similarly, the diatom *Phaeodactylum tricornutum* harbors very few viral genes, despite the fact that many diatom species are known to be virus-infected. However, the retention of giant viral endogenizations may be transient. For instance, among the numerous sequenced strains of the fungus *Rhizophagus irregularis*, only one harbors a 1.5 Mb viral element (Zhao et al. 2023), and retention of endogenous viruses is very scarce across *Amoebidium* or *Acanthamoeba* strains (Sarre et al. 2024; Blais et al. 2025). These examples suggest that while 5mC may increase a genome's capacity to accommodate and silence large foreign insertions, it does not necessarily lead to long-term retention, nor is endogenization an inevitable consequence of retaining this silencing mechanism.

In conclusion, our study provides further evidence that 5mC serves as a critical epigenetic gatekeeper, regulating the incorporation and silencing of foreign DNA in eukaryotic genomes. We propose this is a potentially ancestral role of 5mC in the LECA, or at least it has evolved convergently across many lineages. As we continue to map out 5mC patterns across diverse lineages, it becomes clear that epigenetic silencing is a key process allowing eukaryotes to integrate and control viral and TE insertions, contributing to the dynamic landscape of eukaryotic genome evolution.

## Materials and Methods

### DNMT Search and Phylogenetic Analysis

We gathered a dataset comprising a wide range of eukaryotic proteomes using EukProt database (Richter et al. 2022), complemented with some extra eukaryotic species (see supplementary table S1, Supplementary Material online). Furthermore, we also gathered all the giant virus genomes from Giant Virus DB (Aylward et al. 2021), and all *Mirusvirus* from a previous publication (Gaïa et al. 2023). All Asgardarchaeota genomes were downloaded from NCBI. We used HMMER3 to search these proteomes using the cytosine methyltransferase domain PF00145 HMM profile from PFAM, requiring a *E*-value < 0.0001. The subsequent hits were extracted and all the viral and archaeal hits were collapsed using CD-HIT, clustering sequences with identity above 0.8 (-c 0.8). The resulting sequences were then aligned using MAFFT with the L-INS-i parameter (Katoh and Standley 2013), and trimmed using TrimAL with gappyout mode (Capella-Gutiérrez et al. 2009). The resulting trimmed alignment was then used as input for IQ-TREE2 allowing for automatic model testing (Minh et al. 2020).

All DNMT sequences were then searched using the hmmscan function in HMMER3 to characterize the Pfam-A domain architectures, using an *E*-value threshold of 0.001. The proteomes of *A. castellanii*, *C. paradoxa*, and *N. gruberi* were searched for N6/N4 methyltransferases (PF01555) using HMMER3, retrieving no hits.

### Cell Culture

*A. castellanii* Neff strain was grown at 23 °C in ATCC medium (712 PYG), using 100 mL vented flasks. *N. gruberi* NEG-M was grown at 30 °C in 1,034 Modified PYNFH medium. DNA from untreated cultures of *A. castellanii* and *N. gruberi* was kindly provided by Meritxell Antó of Ruiz-Trillo Lab, Institut de Biología Evolutiva, Barcelona. Purified genomic DNA from *C. paradoxa* was purchased from CCAP collection.

### Treatment With Cytidine Analogues

DNA demethylation drugs 5-Azacytidine (ab142744 Abcam), decitabine (ab120842), and zebularine (ab141264) were dissolved in DMSO as per manufacturer's instructions. *A. castellanii* was grown with 0 M, 10 nM, 100 nM, 1 µM, 10 µM, and 100 µM final concentration of each drug in 4 mL ATCC (712 PYG) 1% DMSO media in a 6 well plate, and effects were tracked daily for 5 d, in duplicate. No treatments displayed a growth phenotype. *A. castellanii* DNA and RNA were extracted from cultures grown for 4 d in 3.5 mL ATCC (712 PYG) 1% DMSO media, with or without their respective drug in triplicate, at a final drug concentration of 100 µM. *N. gruberi* DNA and RNA were extracted from cultures grown under the same conditions, with 1,034 Modified PYNFH media. DNA from drug-treated cultures was extracted using the NEB Monarch Genomic DNA Purification Kit. Total RNA from these cultures was extracted using the Monarch Total RNA Miniprep Kit.

### Methylome Library Construction, Sequencing, and Mapping

For each library, 100 to 200 ng of initial gDNA were spiked in with unmethylated lambda genome control and methylated pUC19 plasmid control. These were sheared to 300 bp using a Covaris sonicator, and then used as input for Enzymatic Methyl-Seq kit (New England Biolabs), following manufacturer instructions. For *N. gruberi* and *A. castellanii*, we also performed Whole Genome Bisulfite Sequencing using the EZ DNA Methylation-Gold Kit (Zymo Research), performing the bisulfite reaction instead of the enzymatic treatment while following the EM-seq protocol. The resulting libraries were sequenced at NovoGene, using Illumina NovaSeq6000 platform, aiming at coverages >30× for reference methylomes, and 5× for shallow treatment samples. The obtained FASTQ files were mapped to the reference genomes (Price et al. 2019; Matthey-Doret et al. 2022; Romero Charria et al. 2024) using BS-Seeker2 (Guo et al. 2013), with BOWTIE2 as backend alignment tool. PCR duplicate reads were removed using Sambamba, and methylation calling was obtained using CGmapTools (Guo et al. 2018). The resulting CGmap files were then imported into R to be analysed using the bioconductor package bsseq. Nonconversion rates for all experiments were obtained computing all C methylation percentage on the lambda genome (∼0.3% in all experiments), while methylation rate at all the CpGs in the pUC19 plasmid were used to control for false negatives (∼98% mCG). Bigwigs of CG and CX methylation were generated using bedGraphToBigWig command from UCSC tools, and then used as input for deepTools (Ramírez et al. 2014) to produce average methylation plots and visualize in the IGV genome browser.

Repeat libraries were obtained from previous studies (Romero Charria et al. 2024), and are based on RepeatModeler2 with LTR module activated (Flynn et al. 2020), then mapped against the genome using RepeatMasker with default parameters. The Kimura distances for each TE insertion were obtained using the RepeatMasker alignments extracting divCpGMod values.

Publicly available Whole Genome Bisulfite libraries were downloaded from NCBI (SRX3236100, SRX22725540, SRX14311868, SRX12841637, SRR042643, and SRR042657) (Zemach et al. 2010; de Mendoza et al. 2018; Bewick et al. 2019; Griess et al. 2023; Hoguin et al. 2023; Sarre et al. 2024) and analysed using the same pipeline. Publicly available Nanopore libraries were downloaded from EBI (S-BSST1363) (Romero Charria et al. 2024). R9 chemistry libraries were basecalled using Guppy, with the dna_r9.4.1_450bps_modbases_5hmc_5mc_cg_sup model to detect both 5mC and 5hmC. The *N. gruberi* R10 library was base called using Dorado, with the 5mC_5hmC sup model (dna_r10.4.1_e8.2_400bps_sup).

Genes were assigned a status of “methylated” or “unmethylated”, using a threshold level of cytosine methylation within gene bodies. The methylation threshold was determined by plotting the distribution of gene body 5mC levels and selecting a value that best separated visibly bimodal distributions, where such patterns were evident. To validate this approach, we applied k-means clustering (*k* = 2) to GBM levels. The average and minimal thresholds obtained through our initial method closely matched those derived from clustering, supporting the robustness of the chosen cut-offs (supplementary fig. S15, Supplementary Material online). No strong sequence-dependent methylation patterns were observed in *N. gruberi*, and so all cytosines were used to calculate methylation levels. In other species, methylation levels were calculated using cytosines in differing nucleotide contexts depending on the genome-wide methylation patterns. *A. castellanii*, *C. paradoxa*, and *P. tricornutum* almost exclusively possess methylation in the mCG context, which was therefore used to calculate methylation levels, while methylation in non-CGC was previously found to mark silenced regions in *Amoebidium* species (Sarre et al. 2024) and mCHG was found to do so in *K. nitens* and *P. patens* (de Mendoza et al. 2018; Domb et al. 2020). In these species these respective contexts were therefore used to calculate methylation levels.

To identify hypermethylated genomic regions in *N. gruberi* and *A. castellanii*, we divided each genome into 500 bp sliding windows with a 250 bp overlap. We then calculated regional methylation averages using the bsseq package in R, and retained all windows with >10% mC. Overlapping windows above this threshold were subsequently merged into continuous regions. These regions were then lifted over to the ENSEMBL genome assemblies of the respective species using LiftOff (Shumate and Salzberg 2021), as the histone ChIP-seq bigWig files from GSE287846 were aligned to those versions (Navarrete et al. 2025). DeepTools2 was used to visualize the histone posttranslational enrichments on hypermethylated regions, using the computeMatrix and plotProfile functions.

### RNA-seq Library Construction, Mapping, and Analysis

We used 200 ng of RNA from treated samples to build mRNA-seq libraries, first enriching for poly-A transcripts with the NEB Magnetic mRNA Isolation Kit S1550S, and then building the libraries with the NEBNext Ultra II Directional RNA Library Prep Kit for Illumina (E7760L) according to manufacturer's instructions. Short read Illumina reads were obtained with a NovaSeq6000 at NovoGene. For *C. paradoxa*, we used publicly available RNA-seq data (SRR8306033) (Gould et al. 2019).

We then used HISAT2 to map RNA-seq reads against the reference genomes (Sirén et al. 2014), to obtain protein-coding gene TPMs using Stringtie, and to perform differential expression analysis of TEs and protein-coding genes, we obtained counts with the TElocal pipeline (Jin et al. 2015). The counts were then analysed with DEseq2 (Love et al. 2014) with default parameters, using DMSO versus treatment comparisons and selecting for genes with a *P*-adjusted value < 0.05 as differentially expressed. Only TEs above 500 bp were kept for the analysis to avoid spurious small repeat annotations. Gene Ontology enrichments were obtained with the TopGO Bioconductor package, using eggNOG-mapper annotations as input (Cantalapiedra et al. 2021).

### Proteome Taxonomic-Origin Assignment

The proteome for each genome annotation was filtered to obtain only the longest/primary isoform per gene, and then DIAMOND [v2.1.9.163 (Buchfink et al. 2021)] was used to search hits against NCBI nonredundant database (version April 2024), obtaining 10 best hits per sequence with an *e*-value cutoff of 0.001. The hits were then assigned taxonomic values using the diamond_add_taxonomy tool (https://github.com/pvanheus/diamond_add_taxonomy). Within-genus hits were discarded from the resulting list (e.g. *Acanthamoeba* hits against *A. castellanii*). The distribution of hits was used to obtain taxonomic categories at the highest level (Eukaryotic, Prokaryotic, Viral and combinations). For *P. patens* genome (Lang et al. 2018), the GEVEs are masked in the V5 genome annotation; therefore, we used ViralRecall (Aylward and Moniruzzaman 2021) with default values to obtain ORFs of GEVE regions.

## Supplementary Material

msaf176_Supplementary_Data

## Data Availability

The data generated for this manuscript has been uploaded to GEO: GSE287846. Code and files to reproduce the analysis presented in this manuscript can be found on this GitHub repository: https://github.com/AlexdeMendoza/Eukaryotic_5mC_Evolution/.
