# Supplementary material for: Repressive Cytosine Methylation is a Marker of Viral Gene Transfer Across Divergent Eukaryotes
Source: Mol Biol Evol. 2025 Jul 25;42(8):msaf176. doi: 10.1093/molbev/msaf176 (PMC12344493; doi:10.1093/molbev/msaf176)
Supplement: msaf176_Supplementary_Data [file msaf176_supplementary_data.zip › SupplementaryFigures.pdf]

Supplementary Material for

"Repressive Cytosine Methylation is a marker of Viral Gene Transfer across divergent eukaryotes"

Luke A. Sarre, Giselle Azucena Gastellou Peralta, Pedro Romero Charria,
Vladimir Ovchinnikov, Alex de Mendoza

Includes Supplementary Figures 1 to 15.

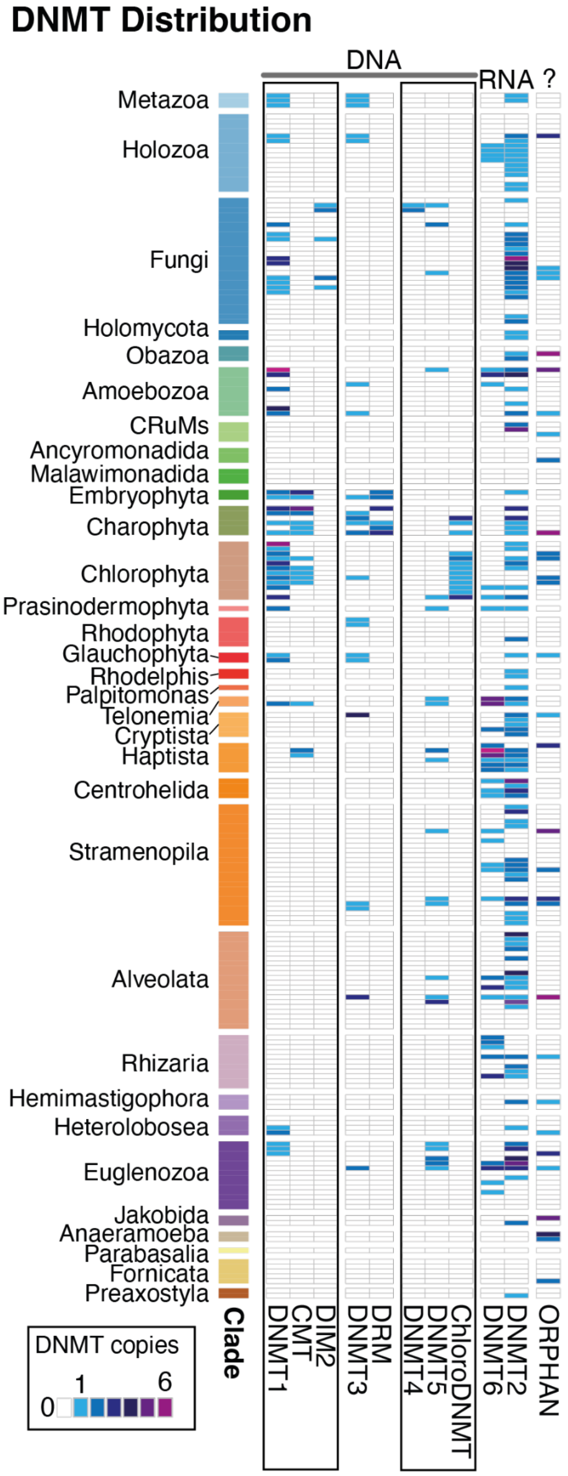

Supplementary Fig. 1. Distribution of DNMT families across the eukaryotes. The presence and absence of families in sampled eukaryotes as sampled in Fig. 1a.

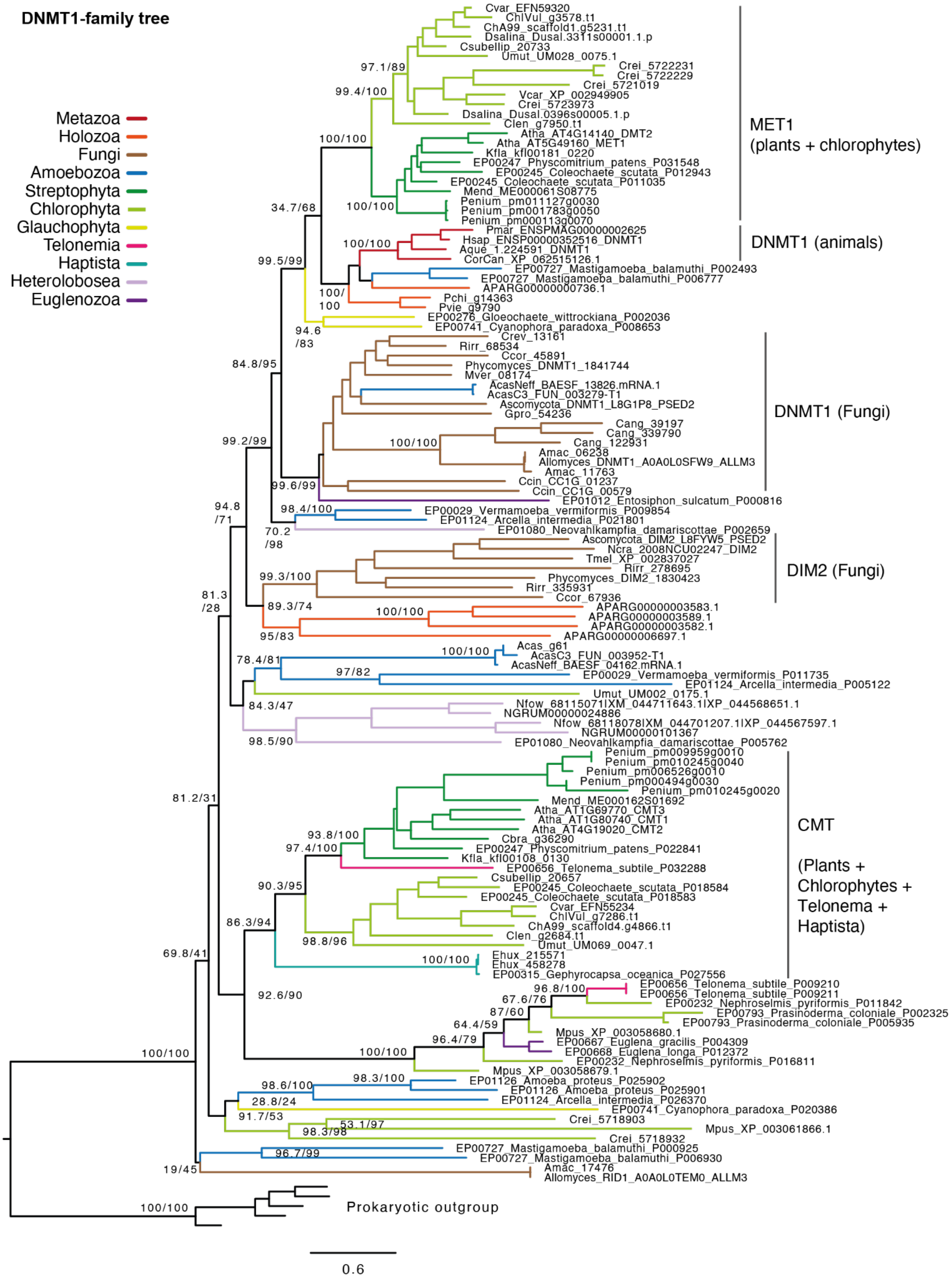

Supplementary Fig. 2. Focused phylogeny of DNMT1-like members. Maximum likelihood phylogenetic tree rooted with the closest prokaryotic outgroups in Fig. 1a for DNMT1 clade sequences. Support values represent 1000 ultrafast bootstrap and aLRT values as computed by IQTREE2. The branch colours are coded as per major eukaryotic groups shown top left.

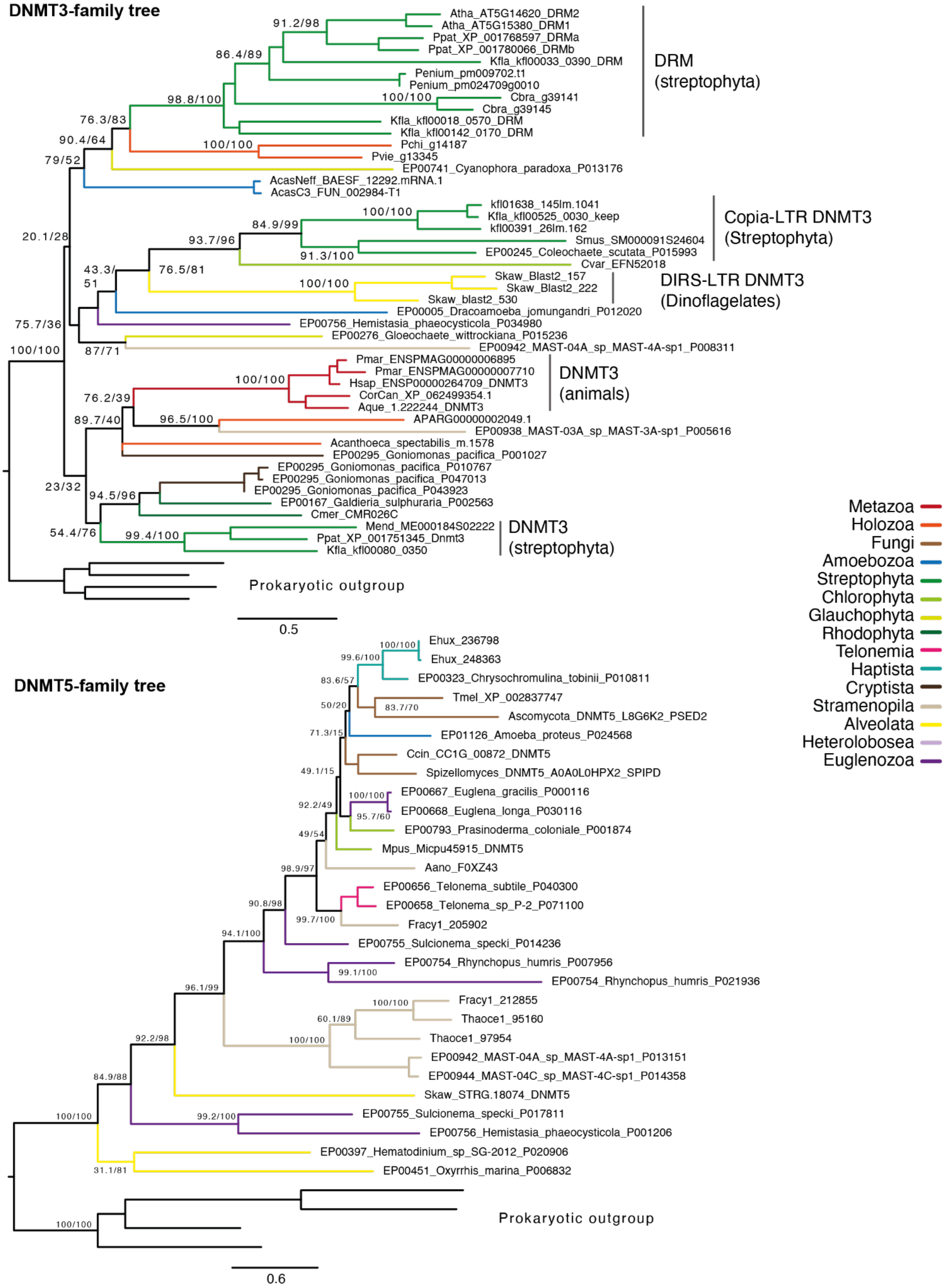

Supplementary Fig. 3. Focused phylogeny of DNMT3 and 5 members. Maximum likelihood phylogenetic trees rooted with the closest prokaryotic outgroups in Fig. 1a for DNMT1 clade sequences. Support values represent 1000 ultrafast bootstrap and aLRT

values as computed by IQTREE2. The branch colours are coded as per major eukaryotic groups shown top left.

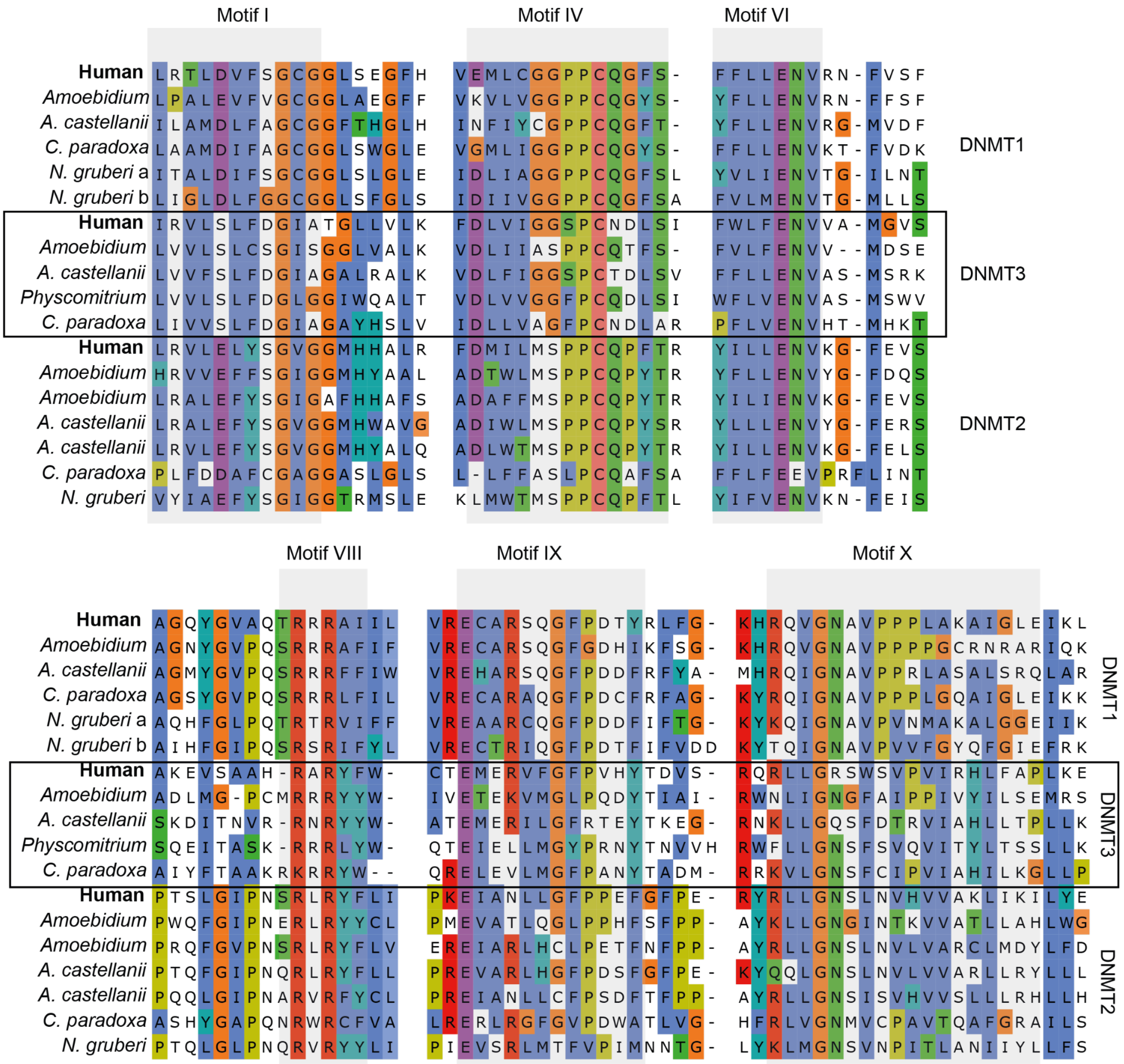

Supplementary Fig. 4. Alignment of DNMT1/2/3 orthologues in *A. castellanii, C. paradoxa* and *N. gruberi*. Multiple sequence alignment of the methyltransferase domain of DNMT1/2/3 orthologues. Alignment performed using MAFFT L-INS-i algorithm. Color coding according to the Clustal palette, methyltransferase functional motifs highlighted with a grey shade. Human DNMT1, DNMT3A and DNMT2 are used as reference sequences.

.

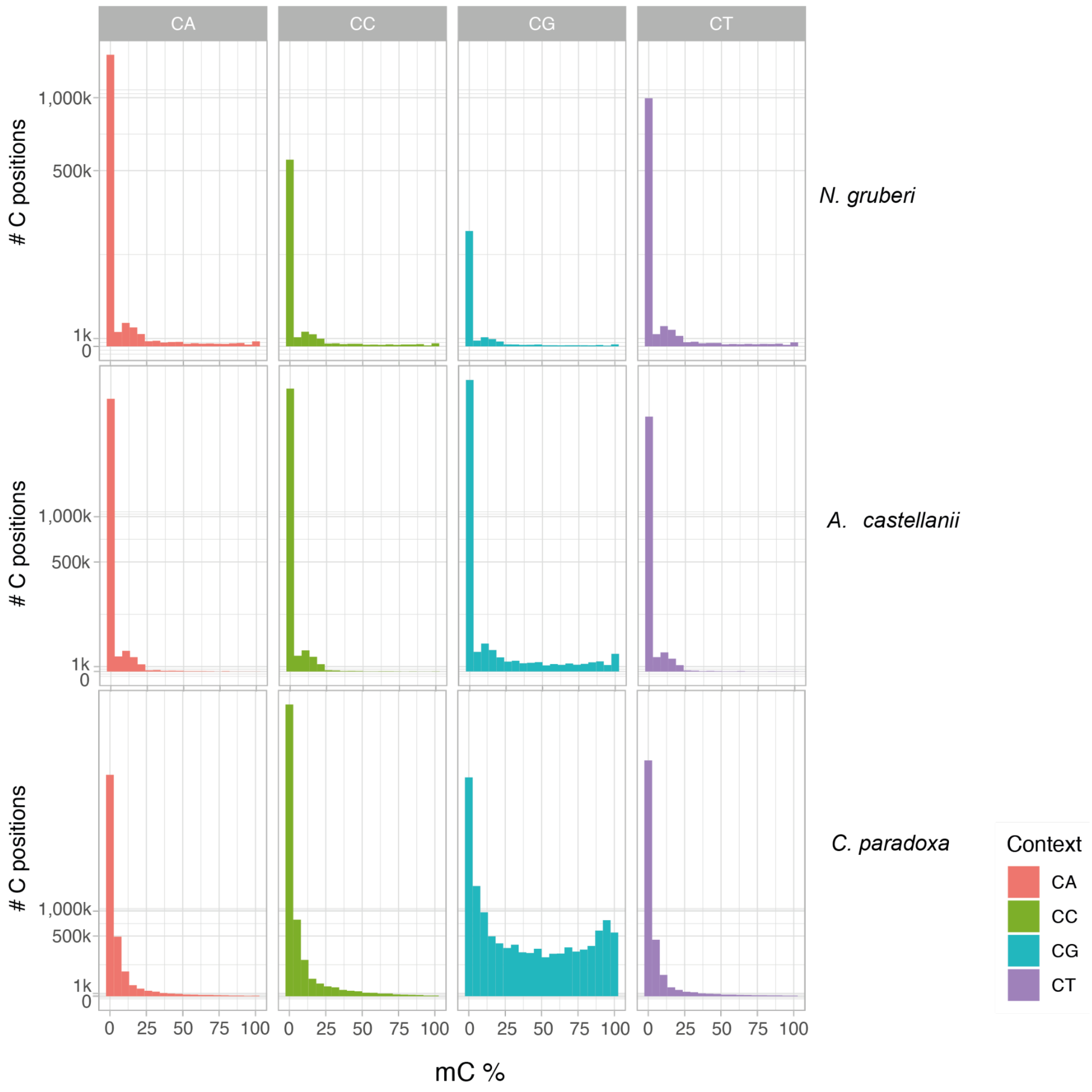

Supplementary Fig. 5. Methylation levels at cytosine resolution. The distribution of per base 5mC levels across all C dinculeotide contexts in *A. castellanii*, *N. gruberi* and *C. paradoxa*. The y axis has been square root converted to highlight the lower counts on *A. castellanii* and *N. gruberi* (ggplot scale_y_sqrt function).

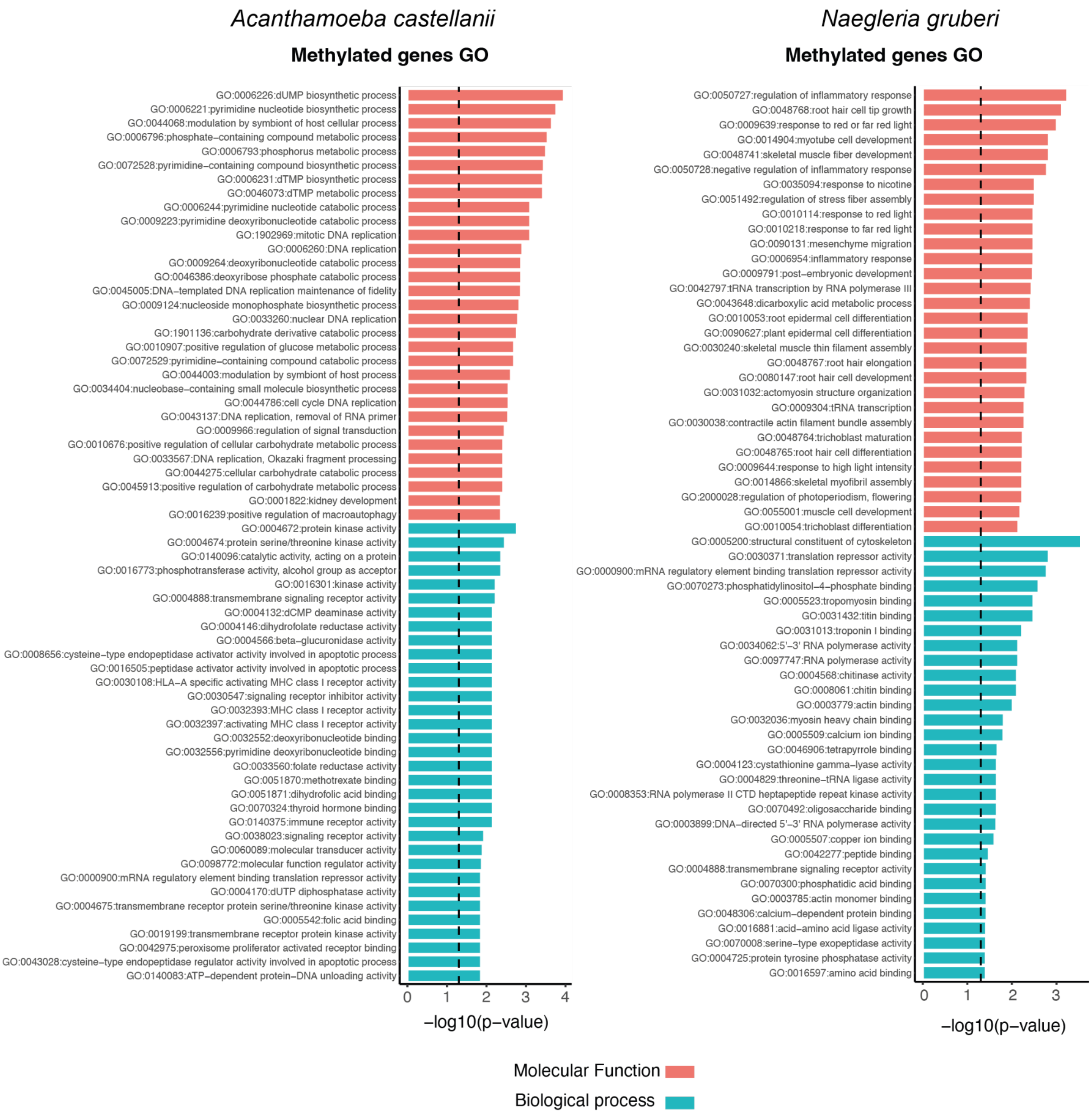

Supplementary Fig. 6. GO functions enriched in *A. castellanii* and *N. gruberi* methylated genes. Gene Ontology enrichments sorted by significance based on Molecular Function (red) and Biological process (green) for the methylated genes. Vertical dashed line indicates p-value of 0.05 according to one-sided Fisher's exact test, as calculated by TopGO.

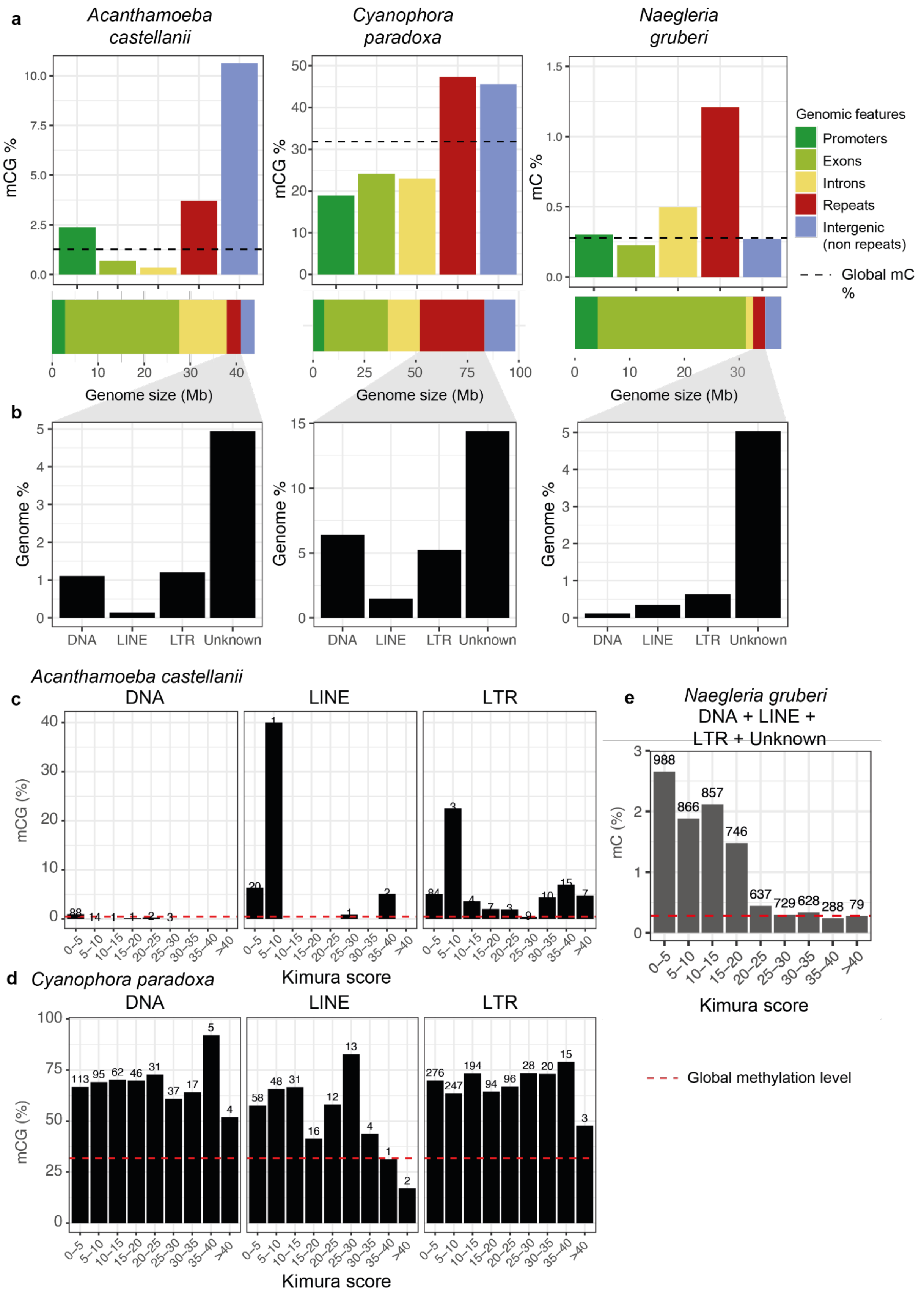

Supplementary Fig. 7. Genome distribution of 5mC in *A. castellanii*, *C. paradoxa* and *N. gruberi*. a) Global methylation levels on various genomic features in the genomes of three unicellular eukaryotes. For *A. castellanii* and *C. paradoxa*, 5mC levels are restricted to the CpG dinucleotide context, whereas for *N. gruberi* is any C. Global methylation levels

across the whole genome are shown as a black dashed line as background. Below, distribution of each genomic feature as proportion of the genome size in each species. **b**) Transposable element composition for each species as per major classes defined by RepeatModeler2 de novo annotation. Global methylation levels on transposable elements arranged per Kimura distance versus consensus sequence, in **c**) *A. castellanii*, **d**) *C. paradoxa*, and **e**) *N. gruberi*. Due to the very low number of classified transposable elements in *N. gruberi*, all repeats are shown together.

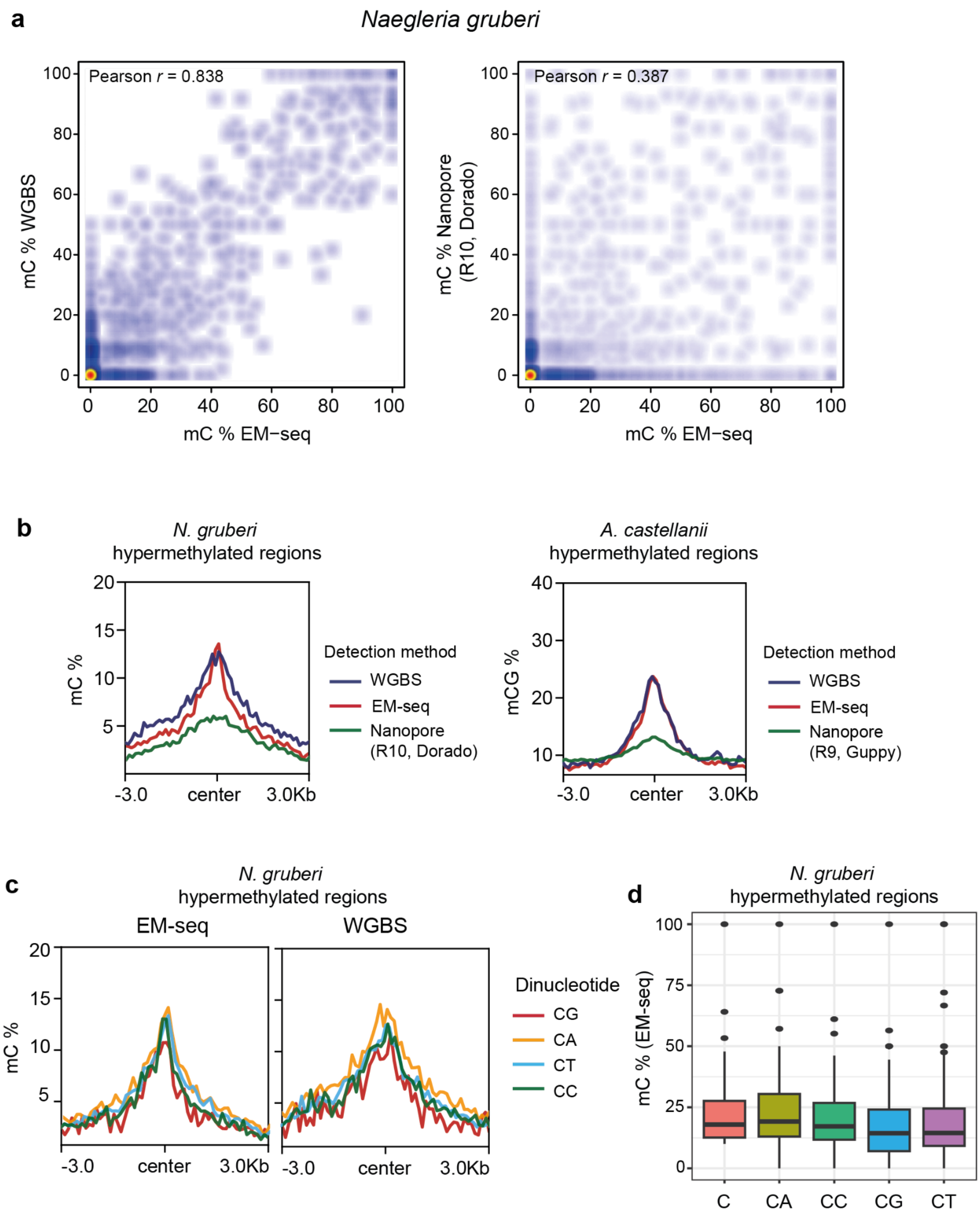

Supplementary Fig. 8. Cross-technology comparison of *N. gruberi* CH methylation. a) Global methylation levels on various genomic features in the genomes of three unicellular eukaryotes

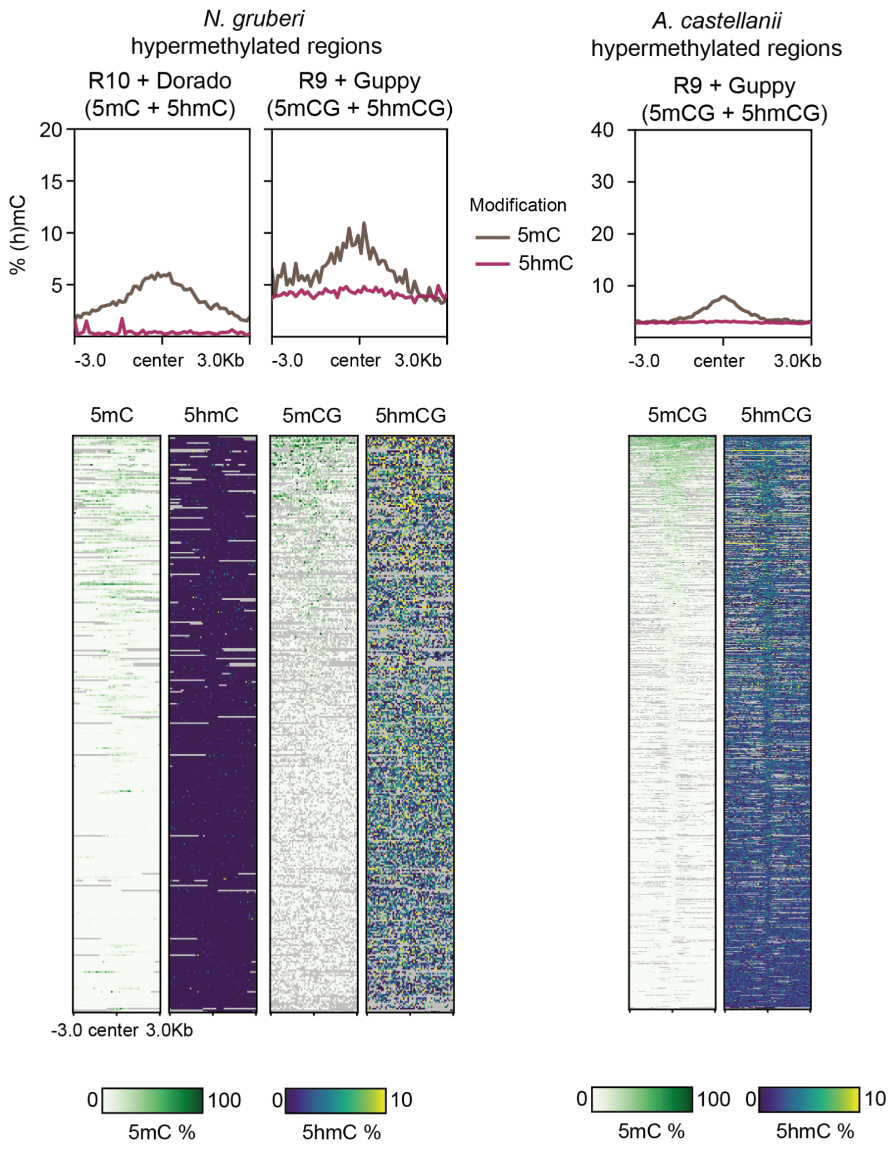

Supplementary Fig. 9. Nanopore signal fails to detect 5hmC enrichment in hypermethylated regions. Hypermethylated regions identified from EM-seq data (Fig. 2) were used to assess the presence of 5hmC using Oxford Nanopore sequencing. For *N. gruberi*, both R9 and R10 datasets were analysed using distinct basecalling algorithms (Guppy and Dorado, respectively) capable of distinguishing between 5mC and 5hmC. *A. castellanii* is included as a control, as it lacks a TET-like enzyme, allowing assessment of background 5hmCG signal in R9-based detection. No consistent 5hmC enrichment was observed in either species.

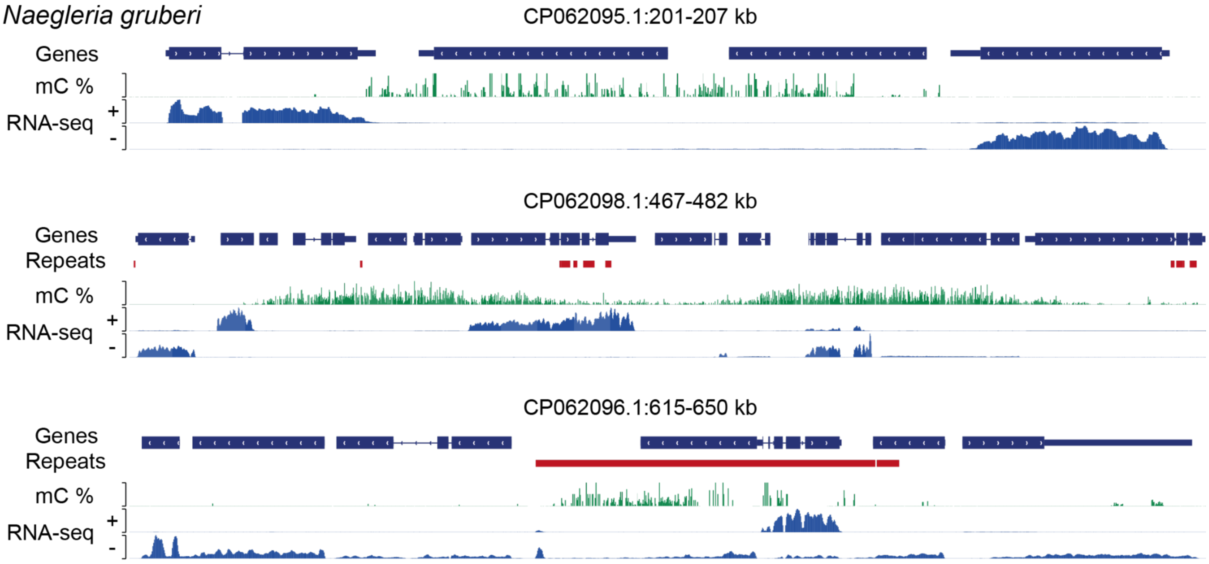

Supplementary Fig. 10. Examples of methylated genomic loci in *N. gruberi*. The relationship between 5mC and transcriptional status across distinct loci and scaffolds, with gene models shown in dark blue, and RepeatMasker repeats shown in dark red. Methylation levels rangeo from 0 to 100%. RNA-seq signal split by stranded information.

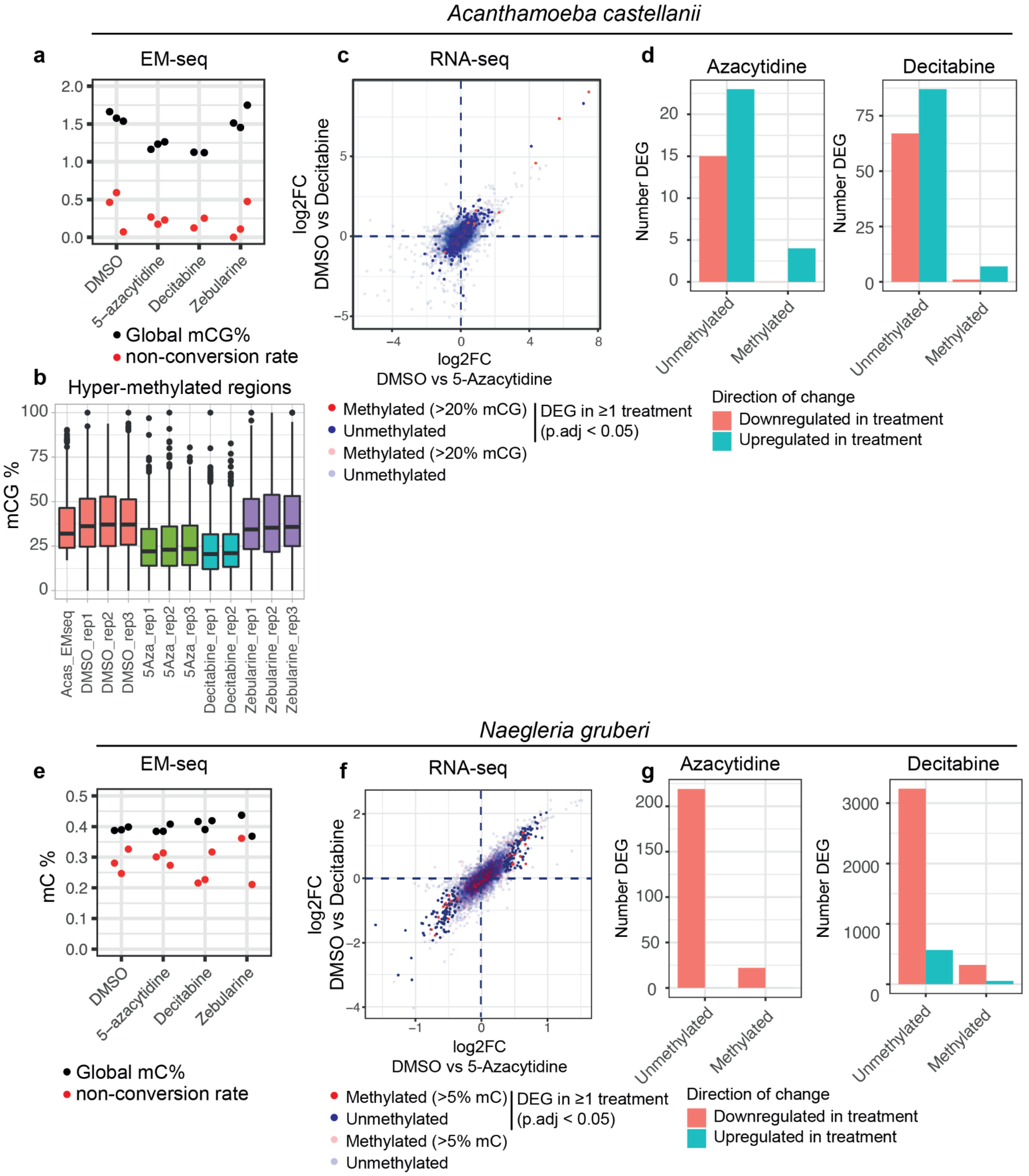

Supplementary Fig. 11. Cytidine analogue treatments of *A. castellanii* and *N. gruberi*. **a**) Global methylation levels in each replicate for each cytidine analogue treatment in *A. castellanii*. **b**) Methylation levels on hypermethylated genomic windows, calculated as >20% mCG 500 bp windows on the reference (untreated) EM-seq dataset. **c**) Differential expression of protein coding genes on *A. castellanii* under 5-Azacytidine and Decitabine treatments vs DMSO control. Red shaded dots indicate methylated genes (>20 % mCG), blue dots indicate unmethylated genes (<20% mCG). Pale dots are non significantly differentially expressed genes (FDR < 0.05) in both treatments. **d**) Number of differentially expressed genes by treatment, classified per methylation status. e) Global methylation levels in each replicate for each cytidine analogue treatment in *N. gruberi*. **f**) Differential expression of protein coding genes on *N. gruberi* under 5-Azacytidine and Decitabine treatments vs DMSO control. Red shaded dots indicate methylated genes (>5 % mC), blue dots indicate

unmethylated genes (<5% mC). Pale dots are non significantly differentially expressed genes (FDR < 0.05) in both treatments. **g**) Number of differentially expressed genes by treatment, classified per methylation status.

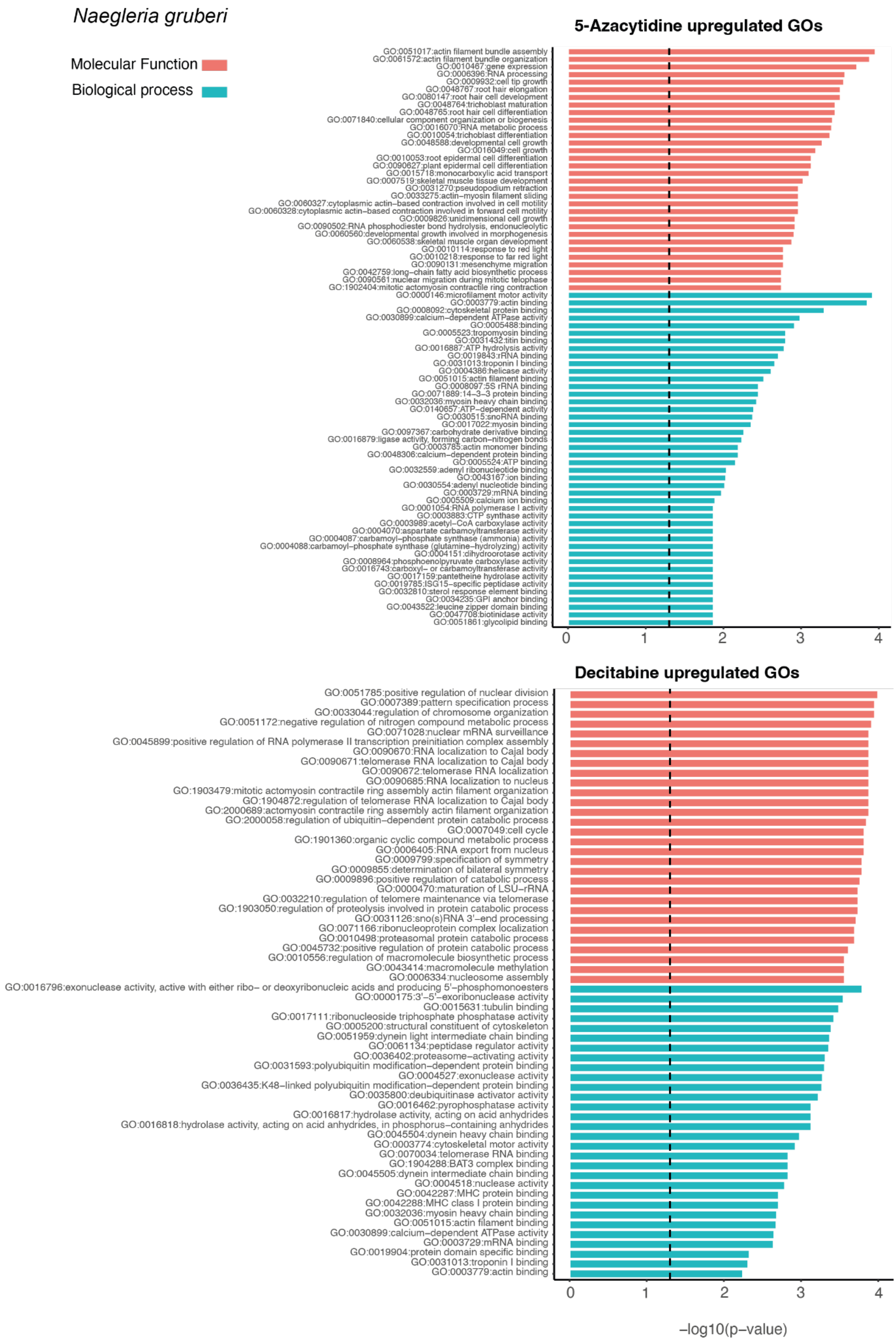

Naegleria gruberi
Molecular Function
Biological process
5-Azacytidine upregulated GOs
GO:0051017:actin filament bundle assembly
GO:0061572:actin filament bundle organization
GO:0010467:gene expression
GO:0006396:RNA processing
GO:0009932:cell tip growth
GO:0048767:root hair elongation
GO:0080147:root hair cell development
GO:0048764:trichoblast maturation
GO:0048765:root hair cell differentiation
GO:0071840:cellular component organization or biogenesis
GO:0016070:RNA metabolic process
GO:0010054:trichoblast differentiation
GO:0048588:developmental cell growth
GO:0016049:cell growth
GO:0010053:root epidermal cell differentiation
GO:0090627:plant epidermal cell differentiation
GO:0015718:monocarboxylic acid transport
GO:0007519:skeletal muscle tissue development
GO:0031270:pseudopodium retraction
GO:0033275:actin–myosin filament sliding
GO:0060327:cytoplasmic actin–based contraction involved in cell motility
GO:0060328:cytoplasmic actin–based contraction involved in forward cell motility
GO:0009826:unidimensional cell growth
GO:0090502:RNA phosphodiester bond hydrolysis, endonucleolytic
GO:0060560:developmental growth involved in morphogenesis
GO:0060538:skeletal muscle organ development
GO:0010114:response to red light
GO:0010218:response to far red light
GO:0090131:mesenchyme migration
GO:0042759:long–chain fatty acid biosynthetic process
GO:0090561:nuclear migration during mitotic telophase
GO:1902404:mitotic actomyosin contractile ring contraction
GO:0000146:microfilament motor activity
GO:0003779:actin binding
GO:0008092:cytoskeletal protein binding
GO:0030899:calcium–dependent ATPase activity
GO:0005488:binding
GO:0005523:tropomyosin binding
GO:0031432:titin binding
GO:0016887:ATP hydrolysis activity
GO:0019843:rRNA binding
GO:0031013:troponin I binding
GO:0004386:helicase activity
GO:0051015:actin filament binding
GO:0008097:5S rRNA binding
GO:0071889:14–3–3 protein binding
GO:0032036:myosin heavy chain binding
GO:0140657:ATP–dependent activity
GO:0030515:snoRNA binding
GO:0017022:myosin binding
GO:0097367:carbohydrate derivative binding
GO:0016879:ligase activity, forming carbon–nitrogen bonds
GO:0003785:actin monomer binding
GO:0048306:calcium–dependent protein binding
GO:0005524:ATP binding
GO:0032559:adenyl ribonucleotide binding
GO:0043167:ion binding
GO:0030554:adenyl nucleotide binding
GO:0003729:mRNA binding
GO:0005509:calcium ion binding
GO:0001054:RNA polymerase I activity
GO:0003883:CTP synthase activity
GO:0003989:acetyl–CoA carboxylase activity
GO:0004070:aspartate carbamoyltransferase activity
GO:0004087:carbamoyl–phosphate synthase (ammonia) activity
GO:0004088:carbamoyl–phosphate synthase (glutamine–hydrolyzing) activity
GO:0004151:dihydroorotase activity
GO:0008964:phosphoenolpyruvate carboxylase activity
GO:0016743:carboxyl– or carbamoyltransferase activity
GO:0017159:pantetheine hydrolase activity
GO:0019785:ISG15–specific peptidase activity
GO:0032810:sterol response element binding
GO:0034235:GPI anchor binding
GO:0043522:leucine zipper domain binding
GO:0047708:biotinidase activity
GO:0051861:glycolipid binding
0
1
2
3
4
Decitabine upregulated GOs
GO:0051785:positive regulation of nuclear division
GO:0007389:pattern specification process
GO:0033044:regulation of chromosome organization
GO:0051172:negative regulation of nitrogen compound metabolic process
GO:0071028:nuclear mRNA surveillance
GO:0045899:positive regulation of RNA polymerase II transcription preinitiation complex assembly
GO:0090670:RNA localization to Cajal body
GO:0090671:telomerase RNA localization to Cajal body
GO:0090672:telomerase RNA localization
GO:0090685:RNA localization to nucleus
GO:1903479:mitotic actomyosin contractile ring assembly actin filament organization
GO:1904872:regulation of telomerase RNA localization to Cajal body
GO:2000689:actomyosin contractile ring assembly actin filament organization
GO:2000058:regulation of ubiquitin–dependent protein catabolic process
GO:0007049:cell cycle
GO:1901360:organic cyclic compound metabolic process
GO:0006405:RNA export from nucleus
GO:0009799:specification of symmetry
GO:0009855:determination of bilateral symmetry
GO:0009896:positive regulation of catabolic process
GO:0000470:maturation of LSU–rRNA
GO:0032210:regulation of telomere maintenance via telomerase
GO:1903050:regulation of proteolysis involved in protein catabolic process
GO:0031126:sno(s)RNA 3'–end processing
GO:0071166:ribonucleoprotein complex localization
GO:0010498:proteasomal protein catabolic process
GO:0045732:positive regulation of protein catabolic process
GO:0010556:regulation of macromolecule biosynthetic process
GO:0043414:macromolecule methylation
GO:0006334:nucleosome assembly
GO:0016796:exonuclease activity, active with either ribo– or deoxyribonucleic acids and producing 5'–phosphomonoesters
GO:0000175:3'–5'–exoribonuclease activity
GO:0015631:tubulin binding
GO:0017111:ribonucleoside triphosphate phosphatase activity
GO:0005200:structural constituent of cytoskeleton
GO:0051959:dynein light intermediate chain binding
GO:0061134:peptidase regulator activity
GO:0036402:proteasome–activating activity
GO:0031593:polyubiquitin modification–dependent protein binding
GO:0004527:exonuclease activity
GO:0036435:K48–linked polyubiquitin modification–dependent protein binding
GO:0035800:deubiquitinase activator activity
GO:0016462:pyrophosphatase activity
GO:0016817:hydrolase activity, acting on acid anhydrides
GO:0016818:hydrolase activity, acting on acid anhydrides, in phosphorus–containing anhydrides
GO:0045504:dynein heavy chain binding
GO:0003774:cytoskeletal motor activity
GO:0070034:telomerase RNA binding
GO:1904288:BAT3 complex binding
GO:0045505:dynein intermediate chain binding
GO:0004518:nuclease activity
GO:0042287:MHC protein binding
GO:0042288:MHC class I protein binding
GO:0032036:myosin heavy chain binding
GO:0051015:actin filament binding
GO:0030899:calcium–dependent ATPase activity
GO:0003729:mRNA binding
GO:0019904:protein domain specific binding
GO:0031013:troponin I binding
GO:0003779:actin binding
0
1
2
3
4
–log10(p–value)

Supplementary Fig. 12. Gene Ontology enrichments of cytidine analogue treatments in *N. gruberi*. GO categories enriched in upregulated genes upon cytidine analogue treatment, sorted by p-value enrichment and divided by molecular function (red) and biological process (turquoise).

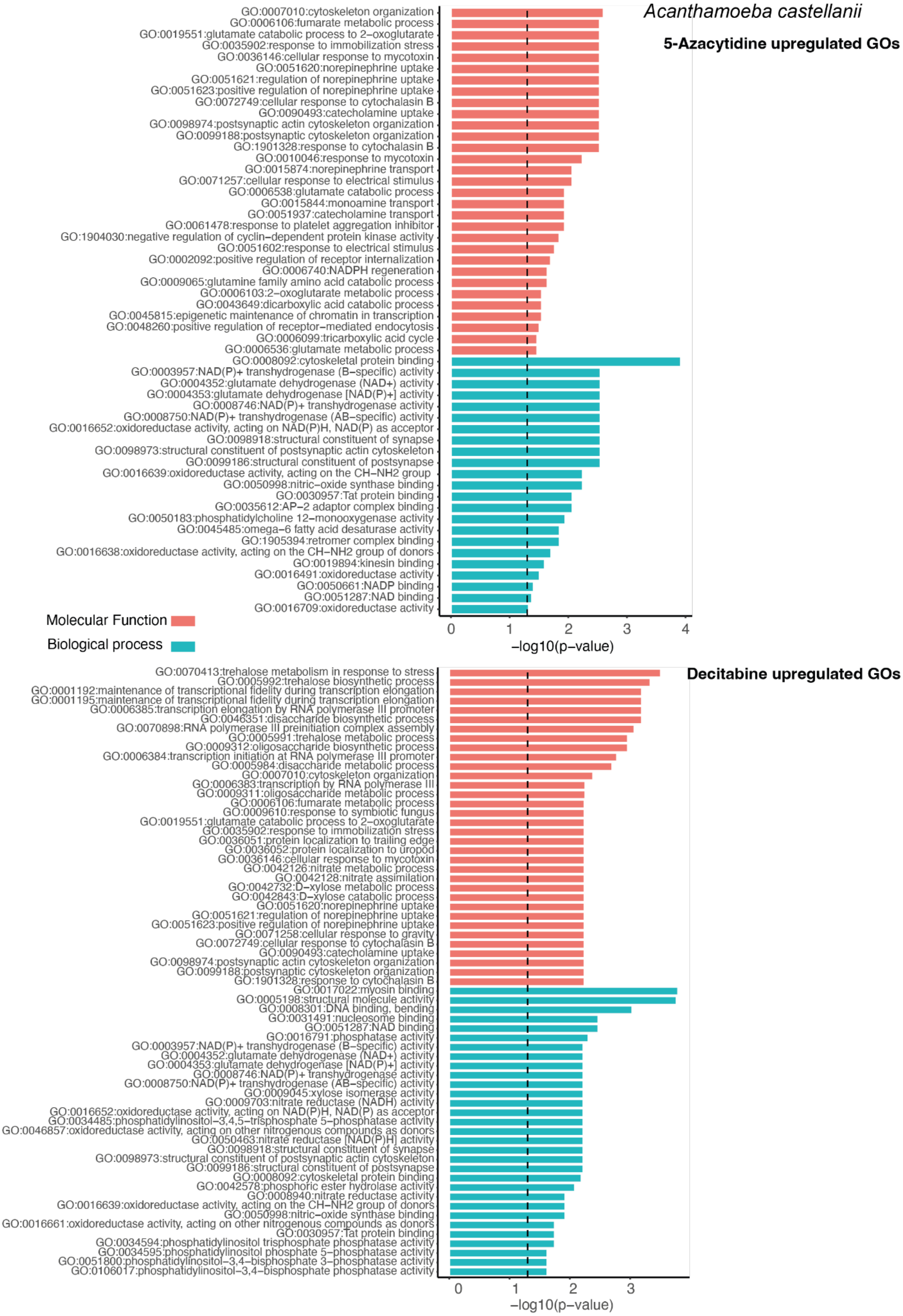

Supplementary Fig. 13. Gene Ontology enrichments of cytidine analogue treatments in *A. castellanii*. GO categories enriched in upregulated genes upon cytidine analogue

treatment, sorted by p-value enrichment and divided by molecular function (red) and biological process (turquoise).

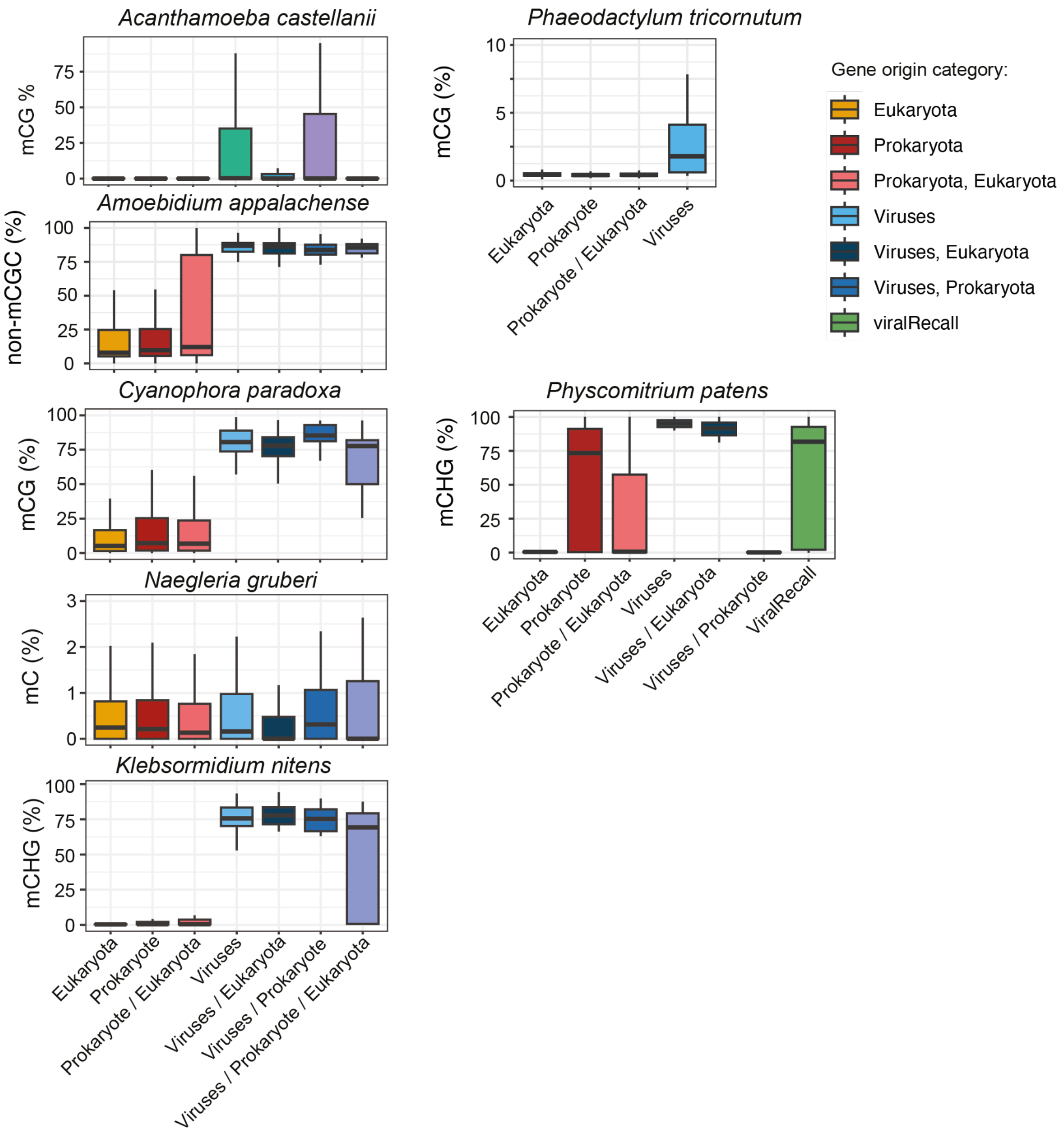

Supplementary Fig. 14. Methylation levels on genes classified as per potential taxonomic origin. Distribution of 5mC levels across 7 species. Taxonomic categories are defined as per DIAMOND searches (see Methods), and 5mC levels are depicted for the sequence context that is associated with silencing in the given species. Center lines in boxplots are the median, box is the interquartile range (IQR), and whiskers are the first or third quartile ± 1.5× IQR.

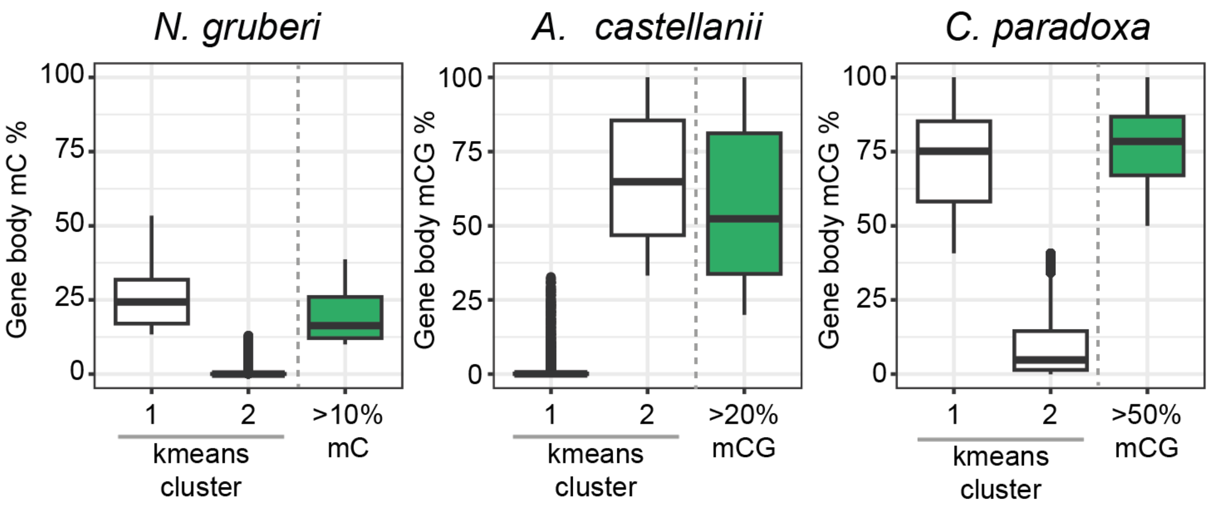

Supplementary Fig. 15. Distribution of gene methylation levels classified using different methods. Methylated genes were defined using two approaches: (1) a species-specific threshold, determined by visual inspection of methylation distributions to capture the peak of highly methylated genes; and (2) k-means clustering (k = 2) of gene body methylation levels. These boxplots compare the methylation level distributions obtained from both methods, showing the separation between clusters 1 and 2 from the k-means analysis, and the placement of species-specific thresholds. The substantial overlap between the two approaches highlights their concordance.
